# Genome of Tetraploid Fish *Schizothorax o'connori* Provides Insights into Early Re-diploidization and High-Altitude Adaptation

**DOI:** 10.1016/j.isci.2020.101497

**Published:** 2020-08-22

**Authors:** Shijun Xiao, Zhenbo Mou, Dingding Fan, He Zhou, Ming Zou, Yu Zou, Chaowei Zhou, Ruibin Yang, Jiaqi Liu, Shilin Zhu, Yajuan Li, Yanchao Liu, Fei Liu, Wanliang Wang, Benhe Zeng, Hong Li, Di Wang, Haiping Liu

**Affiliations:** 1 Institute of Fisheries Science, Tibet Academy of Agricultural and Animal Husbandry Sciences, Lhasa, China; 2 Department of Computer Science, Wuhan University of Technology, Wuhan, China; 3 College of Fisheries, Huazhong Agricultural University, Wuhan, China; 4 College of Fisheries and Life Science, Dalian Ocean University, Dalian, China; 5 Department of Aquaculture, College of Animal Science, Southwest University, Chongqing, China; 6 College of Plant Protection, Jilin Agriculture University, Changchun, Jilin, China; 7 Novogene Bioinformatics Institute, Beijing, China

**Keywords:** Biological Sciences, Evolutionary Biology, Genomics, Transcriptomics

## Abstract

Whole-genome duplications (WGDs) of Schizothoracinae are believed to have played a significant role in speciation and environmental adaptation on the Qinghai-Tibet Plateau (QTP). Here, we present a genome for *Schizothorax o'connori*, a QTP endemic fish and showed the species as a young tetraploid with a recent WGD later than ∼1.23 mya. We exhibited that massive insertions between duplicated genomes caused by transposon bursts could induce mutagenesis in adjacent sequences and alter the expression of neighboring genes, representing an early re-diploidization process in a polyploid genome after WGD. Meanwhile, we found that many genes involved in DNA repair and folate transport/metabolism experienced natural selection and might contribute to the environmental adaptation of this species. Therefore, the *S. o'connori* genome could serve as a young tetraploid model for investigations of early re-diploidization in polyploid genomes and offers an invaluable genetic resource for environmental adaptation studies of the endemic fish of the QTP.

## Highlights

• *Schizothorax o'connori* underwent a recent WGD later than ∼1.23 mya
• Transposon bursts prompted sequence divergence after duplication
• Transposon can induce sequence mutagenesis and regulate gene expression
• Many genes in DNA repair and folate transport/metabolism were naturally selected

## Graphical Abstract

**Figure:**
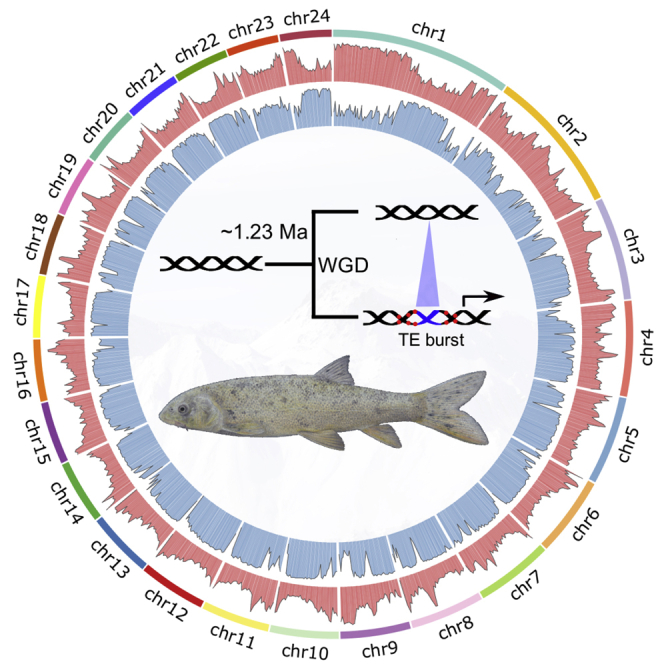

## Introduction

The Qinghai-Tibet plateau (QTP) is the youngest, largest, and highest plateau on Earth, with an average altitude higher than 4,000 m (Pan et al., 2012). The plateau has been uplifting continuously for the past 50 Ma (Jiang and Li, 2014; Li et al., 2015). The high altitude, low temperatures, dramatic temperature fluctuations, and high UV radiation of the QTP make the organisms endemic to this region ideal models for studies of the molecular mechanisms underlying adaptation to harsh environments (Zhang et al., 2016). Schizothoracinae (order Cypriniformes, family Cyprinidae) is an important subfamily of endemic fish on the QTP (Qi et al., 2012), which includes 12 genera and more than 70 species/subspecies (Xu et al., 2016). The overwhelming majority of Schizothoracinae species are polyploid, including examples from tetraploid to hexaploid (Zan et al., 1985), implying that the ancestors of these species underwent the additional subfamily-specific rounds of whole-genome duplication (4R WGD) after the teleost-specific third round WGD (3R WGD). Scientists believed that the WGD of Schizothoracinae played a central role in their speciation and adaptation during QTP uplifts (Zheng et al., 2002). *Schizothorax o'connori* is a typical tetraploid Schizothoracinae species that is distributed widely in the middle section of the Yarlung Tsangpo River, a core area of the QTP (Ma et al., 2010; Zhang, 2011). The complex age structure, slow growth, late sexual maturity, low fecundity, and short reproductive period of this population of *S. o'connori* make them very sensitive to human activities and overfishing (Guo et al., 2016). In a recent investigation of fishery resources in the middle reaches of the Yarlung Zangbo river, it was suggested that *S. o'connori* is a threatened species (Ma et al., 2020). Recent successes in the artificial breeding of *S. o'connori* suggest that this fish may be a promising resource for local aquaculture (Zhang, 2011). Therefore, *S. o'connori* is a potential model for investigations of genome evolution, specifically the re-diploidization process after WGD and the molecular mechanisms underlying high-altitude adaptations of the species of the QTP.

WGD is a major driver of genome variation, as WGD can increase genomic diversity, facilitating possible environmental adaptations and speciation (Berthelot et al., 2014; Lien et al., 2016; MacKintosh and Ferrier, 2017; Xu et al., 2014; Yang et al., 2016). The teleost 3R WGD, which occurred about 320 mya, played an important role in the diversification of ancient teleosts (Yang et al., 2016). Therefore, polyploid fish species can be used to study post-WGD genome evolution and to determine the contribution of WGD to speciation and environmental adaptation (Chen et al., 2019). Re-diploidization is an essential post-WGD evolutionary process, which differentiates and stabilizes duplicated genomes (Lien et al., 2016). The characterization of polyploid genomes might widen our understanding of the post-WGD re-diploidization process and reveal possible mechanisms of speciation and adaptation after WGD. Massive sequence divergences, chromosome rearrangements, and large-scale transposon bursts have been identified as the main features of genome re-diploidization, based on investigations of the *Salmo salar* and *Oncorhynchus mykiss* genomes, which were duplicated 80–103 mya (Berthelot et al., 2014; Lien et al., 2016; Macqueen and Johnston, 2014). However, genome characterizations of the early re-diploidization process of recent WGD, such as the genomic arrangements and transposon expansions along the genome, have not been revealed yet.

In this work, we generated an *S. o'connori* polyploidy genome. We found that *S. o'connori* is a young tetraploid, and its ancestral WGD occurred around 1.23 mya. Although the synteny of the *S. o'connori* genome was largely conserved with respect to that of *Danio rerio*, we detected massive sequence insertion differences between duplicated regions in the *S. o'connori* genome, which are caused primarily by transposon bursts. Those insertions influenced adjacent sequence differentiations and nearby gene expressions. Therefore, we propose that transposon insertions, coupled with the accumulation of mutations and changes in expression patterns in duplicated genes, represent the genomic features of the early stage of re-diploidization after this recent WGD. Meanwhile, a large number of genes associated with DNA repair, folate transport/metabolism, and energy metabolism were positively selected. These genes might participate in the environmental adaptations of *S. o'connori*.

## Results

### Genome Assembly and Annotation

**Figure 1:**
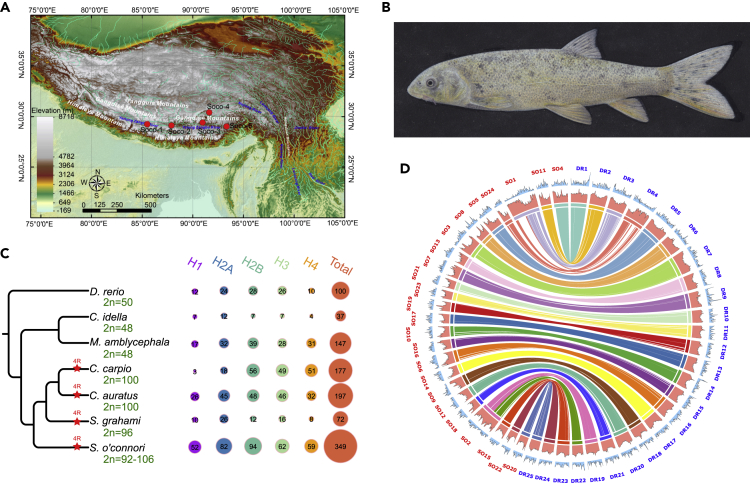
Samples from the Qinghai-Tibet Plateau and Genome Assembly for *Schizothorax o'connori* (A) The sample distribution for the five populations used in this work. (B) A photograph of *Schizothorax o'connori*. (C) The expansion of core histone genes in *Schizothorax o'connori* (family Cyprinidae, subfamily Schizothoracinae), compared with *Megalobrama amblycephala* (family Cyprinidae, subfamily Cultrinae), *Ctenopharyngodon idella* (family Cyprinidae, subfamily Leuciscinae), *Cyprinus carpio* (family Cyprinidae, subfamily Cyprininae), *Carassius auratus* (family Cyprinidae, subfamily Cypriniae), and *Sinocyclocheilus grahami* (family Cyprinidae, subfamily Barbinae). Chromosome numbers are shown in green near species names, and red stars illuminated 4R WGDs. Note that positions of red stars did not represent timings of WGD. (D) The pseudo-chromosome assembly of *Schizothorax o'connori* and conservation synteny relationship to *Danio rerio*.

To collect samples for genome sequencing, wild female *S. o'connori* (see Table S1 for species abbreviations) individuals were captured in the Yarlung Tsangpo river in Tibet (Figures 1A and 1B). The genome of *S. o'connori* was sequenced using next-generation sequencing (NGS) on an Illumina HiSeq XTen platform and using Pacific Bioscience's single-molecule real-time (SMRT) sequencing. We obtained 295.2 Gb of short reads (Table S2) and estimated genome features with a *K-*mer-based method (Table S3). The *S. o'connori* genome is 1.94 Gb, comprising 56.7% repetitive elements. This genome is larger than those of most other teleosts (∼700 Mb) and is similar in size to related genomes that have undergone additional rounds of WGDs, such as *Cyprinus carpio* (1.83 Gb) (Xu et al., 2014) and *Carassius auratus* (1.85 Gb) (Chen et al., 2019). We obtained a coverage depth of approximately 68X, and in total 141 Gb subreads were generated using the Pacific Sequel platform (Table S2). An *S. o'connori* genome of 2.07 Gb length was assembled using FALCON-unzip (Chin et al., 2016) with a contig N50 of 241.9 kb (Tables 1 and S4).

A comprehensive gene prediction strategy, combining *de novo* and homologous proteins-based method, as well as RNA sequencing (RNA-seq)-derived evidence from 15 tissues (Table S5), was performed for the *S. o'connori* genome. The homologous proteins were culled from other Cypriniformes of *Astyanax mexicanus*, *D. rerio*, *Ctenopharyngodon idellus*, *Sinocyclocheilus grahami*, *C. carpio*, and *C. auratus*; a Salmoniforme of *S. salar*; and a Siluriformes of *Glyptosternum maculatum* (Table S6). The common ancestor of the Cypriniforme species is estimated to live about 49–54 mya (Wang et al., 2015). As a result, we identified 43,731 predicted protein-coding genes from the *S. o'connori* genome (Tables 1 and S6). In addition, about 96.4% of all the 2,586 Benchmarking Universal Single-Copy Orthologs (BUSCOs) (Waterhouse et al., 2018) were annotated in the *S. o'connori* genome (Table S7). More than 99.97% of the protein-coding genes predicted in the *S. o'connori* genome could be functionally annotated successfully using public databases (Table S8). This high-quality genome assembly and annotations provide a valuable reference for genome evolution and population genetic studies of *S. o'connori*.

### *S. o'connori* WGD and Chromosomal Comparisons to *Danio rerio*

Previous cytogenetic studies have shown that most species of Schizothoracinae are polyploid (Zan et al., 1985). The karyotype of *S. o'connori* is still controversial and has been reported as 2n = 92 or 2n = 106 in previous studies (Yunfei et al., 1999). The chromosome number is roughly twice as large as diploid cyprinid species, including *D. rerio* (2n = 50) and *C. idellus* (2n = 48) (Wang et al., 2015), suggesting that *S. o'connori* is likely tetraploid (Dai and Han, 2018). Several additional features of the *S. o'connori* genome further support that this genome had been subjected to an extra 4R WGD. First, the number of protein-coding genes predicted from the *S. o'connori* genome (43,731) was comparable to numbers in other tetraploids, such as *C. carpio* (Xu et al., 2014), *C. auratus* (Chen et al., 2019), and *S. grahami* (Yang et al., 2016) (Figure 1C). Second, about 51.2% of BUSCO genes were duplicated (Table S7). Third, there was a clear two-to-one relationship in 25,670 orthologous gene triplets (58.70% of all of the *S. o'connori* genes) between *S. o'connori* and *D. rerio*, which was comparable with those in *C. carpio* (45.83%), *C. auratus* (53.45%), and *S. grahami* (67.74%) (Table S9). Fourth, we identified more than 16 Hox clusters in the *S. o'connori* genome (Figure S1), which was twice the number of Hox clusters of the *D. rerio* genome (Málaga-Trillo and Meyer, 2001).

To identify duplicated contigs in our final assembly, contig sequences were aligned to each other to identify WGD-generated contig pairs, namely, R1 and R2 contigs (Table S10). Our analyses identified 36,255 genes in R1 and R2 contigs and defined 16,264 gene pairs (89.72% of all the genes across both contigs, Table 1) as ohnologs (Berthelot et al., 2014), which suggested that synteny was highly conserved between these duplicated genomes. However, due to their high similarities (see the similarity analysis below), we could not accurately cluster R1 and R2 into two subgenomes using Hi-C (high-through chromosome conformation capture) read mapping. To investigate the genome synteny to other species, we created pseudo-chromosomes by organizing R1 and unpaired contigs using whole-genome Hi-C sequencing data to represent an “averaged” contig order for *S. o'connori* chromosomes. As a result, we constructed 24 pseudo-chromosomes for *S. o'connori* (Figures 1D and S2). We found that these pseudo-chromosomes exhibited a high degree of synteny with the chromosomes of *D. rerio* without obvious inter-chromosomal re-arrangements. A total of 338 collinearity blocks were identified, with an average block size of 3.07 and 4.02 Mb for *S. o'connori* and *D. rerio* (Figure S3), respectively, suggesting that the duplicated genome of *S. o'connori* retained collinear conservations with the *D. rerio* genome after the WGD event.

### Recent Subgenome Divergence for *S. o'connori*

**Figure 2:**
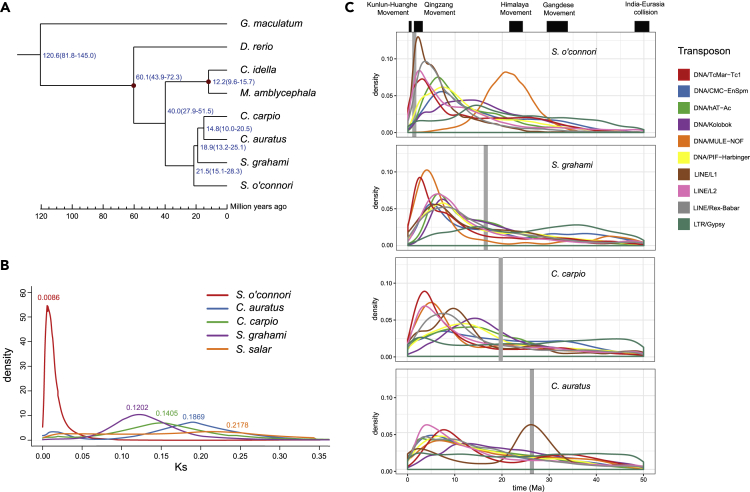
Whole-Genome Duplication and Transposon Burst in the *Schizothorax o'connori* Genome (A) The time estimation for species divergence and whole-genome duplication for *Schizothorax o'connori* and evolutionarily close species. The time is estimated by 95% interval of the estimation. (B) The Ks distribution of ohnologs for tetraploid fish species, including *Schizothorax o'connori*, *Carassius auratus*, *Cyprinus carpio, Sinocyclocheilus grahami,* and *Salmo salar*. (C) The distribution of the insertion time of dominant transposon elements in *Schizothorax o'connori*, *Carassius auratus*, *Cyprinus carpio,* and *Sinocyclocheilus grahami*. The gray bars illuminated the time of subgenome divergence for species.

Ohnologs identified from R1 and R2 sequences were used to determine the divergence time for the subgenomes of *S. o'connori*, *C. carpio*, *C. auratus*, and *S. grahami*. Using the 16,264 ohnologous gene pairs between the R1 and R2 contigs of *S. o'connori*, we identified 30,026 fourfold synonymous third-codon transversion (4DTv) sites. These gene pairs were used to reconstruct a phylogeny of these fish and to estimate divergence times between the sub-genomes of these species. Based on our phylogenetic analysis of orthologous genes after gene clustering in these related fish species (Tables S11 and S12 and Figures S4 and S5), all the fish subjected to 4R WGDs diverged from their most recent common ancestor (MRCA) about 21.5 mya (Figure 2A). The divergence times between *C. carpio* and *C. auratus* were about 10.0–20.5 mya, which was consistent with previous studies (Xu et al., 2014; Yang et al., 2016).

The divergence time for subgenomes was then estimated using the synonymous site substitutions (Ks) from ohnologs. The distribution of ohnolog Ks indicated that the peak Ks for *S. o'connori* subgenomes was 0.0086 (Figures 2B and Table S13), which is significantly smaller than those for other genomes. The smaller Ks value indicated that *S. o'connori* is a young tetraploid compared with other reported natural tetraploid fish species (Figure 2B). On the basis of a Ks rate of 3.51 × 10^−9^ substitutions per synonymous site per year (Xu et al., 2014), we estimated that the divergence time for the subgenomes of *S. o'connori* was about 1.23 mya.

### Dynamics of Transposons in the *S. o'connori* Genome

WGDs are usually closely related to transposon dynamics (Rodriguez and Arkhipova, 2018), because WGDs and transposon bursts occurred simultaneously in many polyploid genomes (Belyayev, 2014). More than 50% of the *S. o'connori* genome is associated with transposons (Table S14). DNA (31.02%) and long interspersed nuclear elements (LINE) (7.6%) were the two most abundant transposons (Table S15). The relative abundance of DNA transposons in the *S. o'connori* genome was lower than that of *D. rerio*, but the relative abundance of LINE elements (XXX%) in the *S. o'connori* genome was significantly higher than those of other cyprinid fish (Tables S16 and S17).

Substitutions identified in a given genome relative to a consensus sequence can indicate the timing of transposon expansions (Albertin et al., 2015). We found that all tetraploid fish underwent a long period of persistent transposon activity along the Himalayan movement, followed by a recent burst around 3 mya (Figures 2C and S6). Although DNA transposons, especially for TcMar-Tc1 and MULE-NOF, mainly contributed to the latest transposon expansion (∼3 mya) in *C. carpio*, *C. auratus,* and *S. grahami*, we observed a significant LINE element expansion, especially for L1, L2, and Rex-Babar, in the recent transposon expansion of the *S. o'connori* genome and L1 expansion with the 4R of *C. auratus* (Figure 2C). Meanwhile, we found that many transposons expanded before the WGD event in *S. o'connori* (Figures 2C and S7), suggesting that the expansion of those transposons occurred in their diploid ancestor lineages.

### Transposon Dynamics May Have Prompted the Re-diploidization of the *S. o'connori* Genome

**Figure 3:**
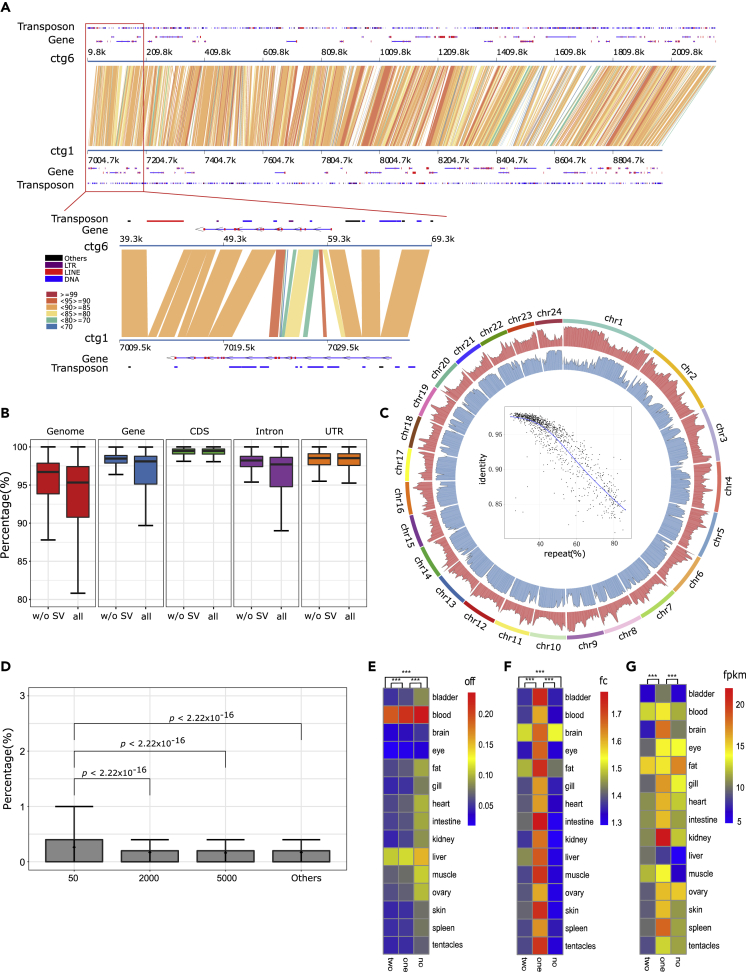
The Sequence Divergence and Structural Variants between Homologous Genomes after the Whole-Genome Duplication in *Schizothorax O'connori* (A) The sequence comparison between ctg1 and ctg6 to illustrate the conserved sequence and transposon insertion after the duplication. (B) The estimated similarity of HSP and regions for whole-genome, gene, coding sequence (CDS), intron, and un-translated regions (UTR). (C) The distribution of sequence divergence and density of transposons along the pseudo-chromosomes for *Schizothorax o'connori*. (D) The SNP density in the adjacent genomic sequences around transposon insertion sites. (E–G) The on-and-off (E), fold changes (F), and FPKM (G) to illustrate the regulation of transposon insertion to the gene expression patterns. Similarities and SNP densities for (B) and (D) are shown in boxplot with error bar for minimal and maximal value. The statistics for (D–G) were performed with Student t test, and ∗, ∗∗, and ∗∗∗ represent statistical p value smaller than 0.05, 0.01, and 0.001, respectively.

The sequence identities of duplicated genome regions reflect the degree of re-diploidization in polyploid genomes (Lien et al., 2016). In the *S. salar* genome that duplicated about 80–103 mya (Berthelot et al., 2014; Lien et al., 2016; Macqueen and Johnston, 2014), only 25.6% of high-scoring segment pairs (HSPs) exhibit more than 90% identity, and the peak HSP identity was 74.9% across the whole genome. However, in the *S. o'connori* genome, 98% of HSPs had more than 90% identity and the peak HSP identity was as high as 97.8% (Figure S8 and Table S18). This was consistent with our aforementioned analysis indicating that *S. o'connori* was a young tetraploid genome. Despite high sequence identities between HSP pairs, we identified 271,251 sequence insertions by direct sequence comparison between duplicated genomic regions (Figure 3A and Table S19). As shown in Figures 3A and 3B, these insertions caused obvious sequence divergence between duplicated genomes, leading to widespread gaps in genome comparisons.

Further analysis showed that the majority of those regions of insertions and deletions (>75%) between duplicated genomic regions were overlapped with transposons, suggesting that the transposon expansion played an important structural role in genomic sequence re-diploidization. More importantly, we found that *S. o'connori* HSP identity was significantly negatively correlated with the local density of transposons (R^2^ = −0.81, p value < 0.001, Figure 3C), which indicated that transposon insertions may have prompted the differentiation of duplicated sequences. Indeed, we found that the mean SNP density within the 500 bases surrounding transposons was significantly higher than the SNP density in remote and random genomic regions (Figure 3D). The enrichments of SNPs around transposon insertion loci implied that transposon insertions might have induced mutagenesis and facilitated the mutation accumulation in nearby genomic regions.

### Transposons May Have Altered the Expression of Duplicated Genes in the *S. o'connori* Genome

Transposon expansions can even alter model genes in a genome (Bourque et al., 2018). We found that roughly 7.8% of all insertions and deletions were located in exon regions of gene loci. Transposon insertions around genes might have influenced the regulation of gene expression and even the sequences and functions of the encoded proteins. To investigate the possible influence of transposon bursts on the regulation of gene expression, the expression patterns of genes with transposons were compared with those of genes without transposon insertions. Based on their genomic coordinates, we categorized ohnologs into three groups, which represented two (T), one (O), and no (N) genes that overlapped with transposons. We first investigated how transposons affected on-and-off gene expression patterns. To this end, the off-gene ratio (number of silenced genes/total number of genes) was calculated for groups in all the tissues tested by RNA-seq. We found that the off-ratios differed significantly between the T, O, and N groups. The off-gene ratio was the lowest in the T group, followed by the O and N groups (Figure 3E), suggesting that transposon insertions could activate the expression of nearby genes. Then, we compared the normalized gene expression (fragments per kilobase million [FPKM]) and fold change (FC) levels within ohnologs between the T, O, and N groups. We found that the FPKM and FC in group O (only one of an ohnolog pair overlapped with a transposon) were significantly higher than those in groups T or N (Figures 3F and 3G).

### Co-expression Patterns of Ohnologs

**Figure 4:**
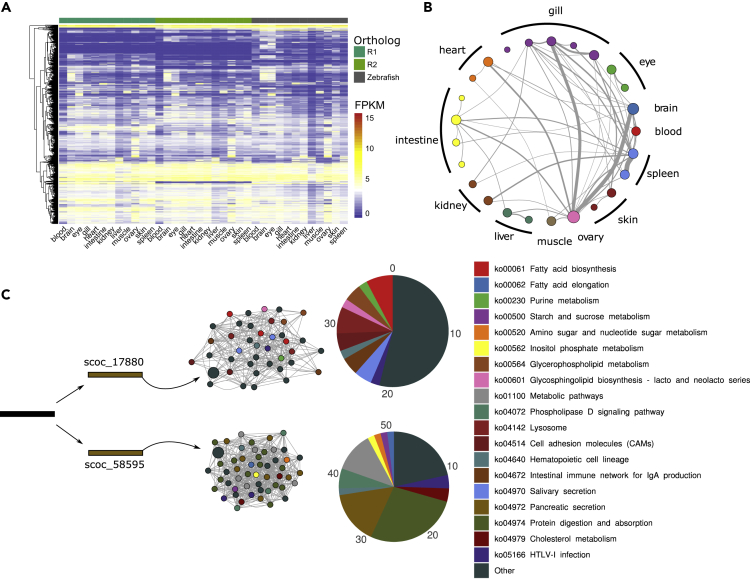
The Sequence and Functional Re-diplodization in the *Schizothorax o'connori* Genome (A) The gene expression heatmap of ohnologs in *Schizothorax o'connori* and *Danio rerio*. (B) The distribution of ohnolog pairs that were involved in distinct co-expression modules. (C) An example for one ohnolog pair of SCOC_17,880 and SCOC_58,595 that was involved in the different co-expression networks and the biological KEGG pathway enrichments for neighboring co-expressed genes.

Gene duplications are important resources for genome evolution and provide potential adaptation for environmental changes because redundancy may alleviate selective pressure and prompt ohnolog differentiation and neofunctionalization (Cheng et al., 2018). The expression patterns of *S. o'connori* ohnologs were analyzed and compared with orthologs from *D. rerio* (Figure 4A, Tables S5 and S20). Based on gene expression profiles in 12 *S. o'connori* tissues, a weighted gene co-expression network analysis (WGCNA) was used to cluster 34,628 genes into 26 functional modules, with an average weight of 0.294 (Figure S9). Using identical types of tissues and RNA-seq data from *D. rerio*, WGCNA clustered 23,122 genes into 20 modules, with an average weight of 0.274 (Figure S10). Based on the correlations between gene models, we assigned expression modules to specific tissues. Interestingly, we identified four and five expression modules that were associated significantly with skin and gill tissues, respectively, which was higher than that of the expression modules from *D. rerio* (Figures 4B and S11). Both gill and skin are essential tissues to sense environmental changes, and we attribute the larger numbers of gene expression modules to possible complicated and elaborate gene expression regulation in gill and skin tissues to external environment factors, such as low temperature of water or UV radiation.

Meanwhile, we identified 232 genes in the core gill expression module that were shared between *S. o'connori* and *D. rerio* (Figure S12). We also identified 843 and 762 species-specific genes in the core gill modules of *S. o'connori* and *D. rerio*, respectively, suggesting that co-expression networks in gill tissues of these two species exhibited obvious differentiation. A Kyoto Encyclopedia of Genes and Genomes (KEGG) pathway enrichment analysis of the species-specific genes for *S. o'connori* gill showed that most of these genes were associated with the adherens junction, vascular endothelial growth factor (VEGF) signaling, and mucin-type O-glycan biosynthesis pathways (Table S21). The adherens junction is needed for both strong cell-cell adhesion and rapid cell-cell contact remodeling during development and wound healing (Meng and Takeichi, 2009). The VEGF signaling pathway is specific to the vascular endothelium, is crucial in both physiologic and pathologic angiogenesis, and may play important roles in the formation of the unique microridges of gill tissues (Eichmann and Simons, 2012; Jing et al., 2004). In addition, roles for mucin-type O-glycans have been well documented for many cell surface and secreted proteins that modulate recognition, adhesion, and communication events occurring between cells and their surrounding environments (Tran and Ten Hagen, 2013). Mucin-type O-glycan biosynthesis generates the primary constituents of mucins that are expressed on various mucosal sites of the body (Jin et al., 2015), and they may play an important role in protecting fish from mechanical injury in rapid flow.

Remarkably, we also found that over 3,000 ohnologs were separated into different expression modules, especially between gill, brain, spleen, and ovary modules (Figure 4B). Genes from one ohnolog pair clustered in different modules might reflect functional divergences in duplicated genes. One example was the ohnologs of the neurotrypsin genes (SCOC_17,880 and SCOC_58,595). These genes were found in different expression modules, and functional analysis revealed that genes with 1° connections to SCOC_17,880 and SCOC_58,595 in the co-expression network enriched into entirely different biological pathways (Figure 4C). However, the mechanism of functional divergence of duplicated genes and their contribution to environmental adaptation and speciation for Schizothoracinae need further gene functional analysis.

### Positively Selected Genes for Environmental Adaptation

Genes with more non-synonymous substitutions than synonymous substitutions (i.e., Darwinian selection) were termed positively selected genes (PSGs). To identify signals of positive selection compared with other fish species, we identified 177 PSGs (Table S22) from *S. o'connori* in the genome using codeml with the branch site model in PAML (Yang, 2007). Our functional analysis showed that cell-cell adhesion, DNA repair, immune response, Fanconi anemia pathway, and the phagosome were all enriched in PSGs (Tables 2, S23, and S24). To identify natural positive selection between ohnologs, we compared ohnolog sequences using the KaKs_Calculator (Zhang et al., 2006). As a result, we identified 503 pairs of positively selected ohnologs (Table S25). An enrichment analysis of those genes showed that cell-cell adhesion, mismatched DNA binding, mismatch repair, nucleosome assembly, and homologous recombination were over-represented (Tables S23 and S24). Meanwhile, we also identified 8.6–8.8 million SNPs in the *S. o'connori* genome from 199 individuals in five populations (Figures 1A and Tables S26–S28). Selective sweep analysis was performed using whole-genome resequencing data from different high-altitude populations (3,100–4,700 m) (Table S26), and 194 genomic regions that harbored 306 PSGs (Table S29) were determined to exhibit different genetic diversity among these populations. Interestingly, those genes were enriched in fatty acid biosynthesis, mucin-type O-glycan biosynthesis, and ion channel activity.

## Discussion

In this work, we present a reference genome for *S. o'connori* using long read data from PacBio sequencing. The continuity of the genome was assessed, and the contig N50 length of the *S. o'connori* genome was substantially greater than those of the previously constructed *O. mykiss* (7.7 kb), *S. grahami* (29.3 kb), *S. salar* (57.6 kb), and *C. carpio* (68.4 kb) genomes (Berthelot et al., 2014; Lien et al., 2016; Xu et al., 2014; Yang et al., 2016; Chen et al., 2019). We also noticed that the contig N50 of *C. auratus* (816.8 kb) was higher than that of *S. o'connori* (Chen et al., 2019). The *C. auratus* genome was also assembled using long reads, and the allopolyploid nature of *C. auratus* genome may have facilitated the sequence assembly of subgenomes. To further evaluate the quality of assembly, high-quality NGS short reads were aligned to our assembled genome. More than 98% of all the reads were properly mapped upon the genome, and only 0.0042% of detected SNPs were homozygous, indicating that the genome sequence was highly accurate at the base level (Table S30). In addition, the completeness of the genome was evaluated by BUSCO (Waterhouse et al., 2018) and the core eukaryotic genes mapping approach (CEGMA) (Parra et al., 2007). As a result, 2,586 BUSCO orthologs were present in the genome, of which 94.9% and 2.7% were completed and partial, respectively (Table S7). A total of 97.6% of the 248 core eukaryotic genes in the genome were also detected by CEGMA (Table S31).

Using gene annotation of the *S. o'connori* genome, we observed that the core histone genes, which included H1, H2A, H2B, H3, and H4, were significantly expanded in the *S. o'connori* genome, compared with other cyprinid species (Figure 1C). Histone genes play important roles in DNA packing and structural stability (Mariño-Ramírez et al., 2005), response to stress (e.g., to cold, Verleih et al., 2015), antibiotic stimulation (Lü et al., 2014; Noga et al., 2011), UV radiation (Pawlak and Deckert, 2007), and DNA-damage repairing (Schild-Poulter et al., 2003). The expanded histone genes might participate in genome stability and could contribute to adaptations to the harsh environments of cold stress, UV radiation, and DNA damage. However, the biological significances of these expanded histone genes in *S. o'connori* need further functional and evolutionary investigations.

The molecular mechanism of WGD in *S. o'connori* was not clear up to now. Although many tetraploid fish species in Cyprinidae lineages, such as *C. carpio* and *C. auratus,* are typical allotetraploid, *S. o'connori* exhibited obvious distinct genome features compared with *C. carpio* and *C. auratus*. First, a phylogenetic analysis showed that sugbenomes of *C. carpio* and *C. auratus* were clustered into two clades across two species, indicating one *C. carpio* subgenome was evolutionarily closer to one *C. auratus* subgenome. However, the subgenomes of *S. o'connori* were clustered into a single clade (Figure S13). This result was consistent with the sequence identity between subgenomes of ∼97.8% (Figure S8) and the divergence time of subgenomes for *S. o'connori* of 1.23 mya, which was much more recent than the divergence time of 14.4 mya for *C. carpio* and *C. auratus*. Second, we performed transposon analysis for homologous genomic regions. Transposons inserted into the same loci of homologous genomic regions should be ancient transposon duplications in diploid ancestors. We found that more than 50% of transposons were ancient in duplicated genomic regions (Table S32) and exhibited good synteny (Figure S14). In addition, we also performed a transposon family phylogenetic analysis to clarify the WGD origin of sterlet sturgeon (Du et al., 2020). We found that transposon families, including DNA/TcMar-Tc1, DNA/hAT-Ac, LINE/L2, and LINE/Rex-Babar, in the *S. o'connori* genome were monophyletic (Figures S15–S18), exhibiting no evidence of an allopolyploid WGD. Therefore, our genomic analysis showed that the *S. o'connori* polyploidization was probably formed by autopolyploidization. However, this conclusion needs further confirmation by experiments such as cytogenetic studies.

We estimated 1.23 mya for the subgenome divergence time for *S. o'connori*. As we discussed, if *S. o'connori* was autotetraploid, the WGD of *S. o'connori* occurred around 1.23 mya. However, if *S. o'connori* was allotetraploid, the parental genomes were separated about 1.23 mya, and the 4R WGD of *S. o'connori* occurred even later. Regardless, *S. o'connori* is a young tetraploid compared with other reported natural tetraploid fish species, making this species a potential model for investigating the early post-WGD genome evolution and re-diploidization process. Meanwhile, the estimated subgenome divergence time was also coincident with the recent multiple rounds of uplift and deplanation of the QTP during the Tertiary period, leading to the overall height of the final plateau to ∼3,000 to 3,500 m (Jiang and Li, 2014). In parallel with these plateau uplifts, Pleistocene glaciation began about 2.58 mya (Gibbard et al., 2010). The harsh environments jointly caused by these rapid QTP uplifts and Quaternary glaciation, including the arctic condition, nutritive deficiency, and high levels of UV exposure, might have been dominant factors driving the 4R WGD of *S. o'connori*. Interestingly, the historical effective population size of *S. o'connori* also declined from 1 to 2 mya but remarkably increased after about 0.9 mya (Figure S19). These results suggested that the ancestor of *S. o'connori* may have undergone a bottleneck effect after the rapid QTP uplifts and Quaternary glaciation, and a population expansion afterward (Figure S19).

We showed here that a genome-wide transposon burst occurred in the *S. o'connori* genome. Those transposons may have played important structural and functional roles in the genome evolution of *S. o'connori*. Our results showed that more than 75% of insertion and deletion differences between duplicated genomic regions were related to transposon elements (Figure 3A). Meanwhile, we analyzed base compositions around insertion sites and found an obvious TA-enriched pattern (Figure S20). Notably, the TA motif is a typical insertion site for DNA transposons, further confirming the contribution of transposon bursts to these sequence divergences. In addition, our gene expression analysis implied that asymmetric transposon insertions may have caused a differentiation in gene expression between ohnologs. These results illuminate that transposon insertions not only could have induced sequence mutagenesis between duplicated genomes but also prompted the differentiation of gene regulation and expression in ohnologous gene pairs.

WGD events double the number of genome-wide functional genes, generating massive redundant genes (Van de Peer et al., 2017). The duplication and expansion of functional genes may be crucial for environmental adaptation and species survival (Alix et al., 2017). Becoming a singleton is one fate of duplicated genes and might play an important role in maintaining cellular functions. Interestingly, we found that central genes in the main crossover pathway (ZMM pathway), namely, *msh4* and *msh5*, were both singletons. *Msh4* and *msh5* are important non-homology recombination suppressors during meiosis, and a singleton of *msh4* prevents non-homologous crossovers in the polyploid *Brassica napus* (Gonzalo et al., 2019). Therefore, these singleton genes in the ZMM pathways may have contributed to the stability of the *S. o'connori* genome after WGD, along the whole-genome of *S. o'connori*, Based on the ordering of genes in the synteny blocks between homologous genomic regions (Figure S21), we found that about 10.99% of singletons could be identified using gene synteny information, which is slightly lower than that in *C. auratus* (11.7%) and *S. grahami* (18.59%) (Table S33). Considering that the WGD in the S. o'connori genome is much more recent, if *S. o'connori* is autotetraploid, our results showed that *S. o'connori* might have experienced rapid gene loss after the 4R WGD. However, if *S. o'connori* is allotetraploid, the gene loss might have occurred from the parental genomes.

**Figure 5:**
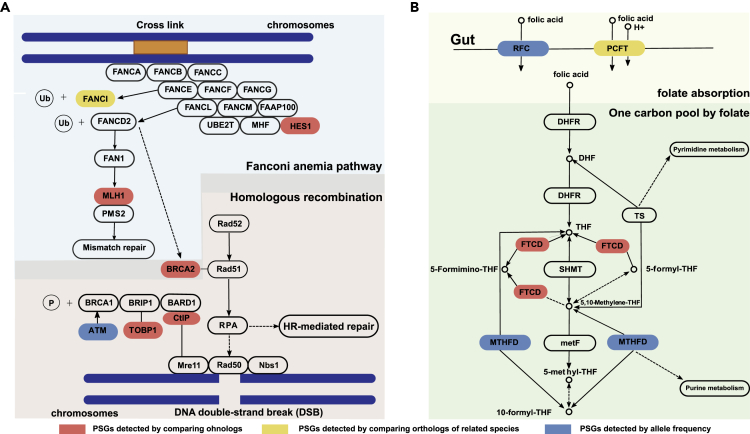
The Pathway that Illustrates the Positively Selected Genes in Folate Metabolism and DNA Repairs in *Schizothorax o'connori* (A and B) The positively selected genes involved in folate transport and metabolism (A) and pathway for homologous recombination and fanconi anemia pathway (B). Genes that were positively selected from ohnolog pair comparisons and codon-, and population-based methods highlighted in red, yellow, and blue, respectively.

We also identified putative naturally selected genes for *S. o'connori* through ortholog, ohnolog, and population comparisons (Tables 2, S22, S25, and S29). We performed functional analyses for PSGs based on various methods separately. Notably, many PSGs identified from ortholog and ohnolog comparison were involved in the biological function of DNA repair, cellular response to DNA damage stimulus, fanconi anemia, homologous recombination, and immune response (Table 2 and Figure 5A). In addition to the identified selected genes in aforementioned biological functions, a population comparison also detected many PSGs contributing to folate and energy metabolism (Table 2 and Figure 5B).

**Table 1:** Assembly Statistics of the *Schizothorax O'connori* Genome and Annotations

	Genome Assembly
Total length (bp)	2,067,143,420
Sequence number	25,126
Max sequence	9,078,463
Number ≥ 2000	24,148
N50 length	241,912
Gene number	43,731
Singletons	3,899
Ohnologs	25,670

**Table 2:** The Putative Positive Selected Function Genes in *Schizothorax o'connori* Genome for DNA Repairing, Folate Transport and Metabolism, and Energy Metabolism

ID	Symbol	Method	Pathway	Type
SCOC_29972	*brca2*	Ohnologs	Fanconi anemia pathway, homologous recombination	I
SCOC_48653	*topbp1*	Ohnologs	Homologous recombination	I
SCOC_29848	*Atm*	Population	Homologous recombination	I
SCOC_41124	*tdp1*	Codon	DNA repair	I
SCOC_43553	*igfbp3*	Population	p53 signaling pathway: insulin-like growth factor-binding	I
SCOC_43551	*igfbp1a*	Population	p53 signaling pathway: insulin-like growth factor-binding	I
SCOC_25202	*mthfd1b*	Population	One-carbon pool by folate	II
SCOC_39892	*mthfd1b*	Population	One-carbon pool by folate	II
SCOC_42360	*slc19a1*	Population	Vitamin digestion and absorption	II
SCOC_23906	*Ftcd*	Ohnologs	One-carbon pool by folate	II
SCOC_17594	*hmgcl*	Population	Synthesis and degradation of ketone bodies	III
SCOC_47651	*abat*	Codon	Carbohydrate metabolism: Butanoate metabolism, propanoate metabolism	III
SCOC_55492	*Hyi*	Codon	Carbohydrate metabolism: Glyoxylate and dicarboxylate metabolism	III
SCOC_03692	*plpp4*	Population	Lipid metabolism: Glycerophospholipid metabolism, glycerolipid metabolism	III
SCOC_17661	*etnppl*	Population	Lipid metabolism: Glycerophospholipid metabolism	III
SCOC_30552	*adprm*	Population	Lipid metabolism: Glycerophospholipid metabolism	III
SCOC_45459	*dgke*	Population	Lipid metabolism: Glycerophospholipid metabolism, glycerolipid metabolism	III
SCOC_15952	*dgat1a*	Codon	Lipid metabolism: Glycerolipid metabolism	III
SCOC_50230	*gk3p*	Codon	Lipid metabolism: Glycerolipid metabolism	III
SCOC_39893	*gpx1a*	Population	Lipid metabolism: Arachidonic acid metabolism	III
SCOC_46334	*cyp2ad2*	Population	Lipid metabolism: Arachidonic acid metabolism, linoleic acid metabolism	III
SCOC_11670	*degs1*	Population	Lipid metabolism: Sphingolipid metabolism	III

Types I, II, and III denote possible PSGs in the DNA repairing, folate transport and metabolism, and energy metabolism, respectively.

Homologous recombination and fanconi anemia pathway play an important role in the repair of double-stranded DNA and the removal of DNA interstrand cross-links (ICLs) (Li and Heyer, 2008; Rodríguez and D'Andrea, 2017). The deleterious UV radiation exposure caused by rapid plateau uplifts might damage double-stranded DNA and induce ICLs (Cecchini et al., 2005). The *brca2* gene, crucial for the repair of damaged DNA and for stably maintaining cellular genetic information (Wooster et al., 1995), was identified from ohnolog comparisons (dN/dS of 1.08) (Figure 5A). The *tpd1* gene, participating in the repair of free-radical-mediated DNA double-strand breaks (Dong et al., 2012), was identified using a population comparison. The natural positive selection of key genes that participate in DNA repair might contribute to the maintenance of integrity of genetic information in the *S. o'connori* genome when exposed to intense UV radiation and dramatic temperature fluctuations.

We also identified three PSGs in the folate transport and metabolism pathways. Solute carrier family 19 member 1 (*slc19a1*) and methylenetetrahydrofolate dehydrogenase (*mthfd*) were identified from a population comparison, and formiminotetrahydrofolate cyclodeaminase (*ftcd*) was identified from an ohnolog comparison (dN/dS of 1.35) (Figure 5B). Folate is a vitamin involved in the one-carbon transfer reaction, DNA synthesis, and methylation (Lucock, 2000). In addition, a previous association study of high-altitude populations revealed that methylenetetrahydrofolate reductase (*mthfr*) was selected in Tibetans, indicating the importance of folate in UV protection and DNA damage repair after UV exposure (Yang et al., 2017). Folate is vulnerable to degradation by UV radiation (Steindal et al., 2007), and increased exposure to solar UV radiation reduces the efficacy of folic acid supplementation (Borradale et al., 2014). *Slc19a1* plays a crucial role in folate absorption (Chiao et al., 1997) and intracellular folate concentration regulation (Hou and Matherly, 2014). The genes *mthfd* and *fctd* are important for folate metabolism and in the “one-carbon pool by folate” pathway for DNA biosynthesis (Watkins and Rosenblatt, 2012), and *mthfd* is also associated with gastric carcinogenesis (Wang et al., 2007) and Down syndrome (Scala et al., 2006). Natural selective pressure on *mthfd* and *fctd* might improve folate metabolism and DNA synthesis after exposure to UV radiation in *S. o'connori*.

Ketone bodies have been implicated not only in energy metabolism but also in lipogenesis (Chavan et al., 2016). We found that 3-hydroxy-3-methylglutaryl-CoA lyase (*hmgcl*), a key gene in ketogenesis and metabolism regulation during fasting or starvation (Kang et al., 2015), was under positive selection in populations living at different altitudes. This gene might play an important role in the environmental adaptation of *S. o'connori* to nutrient deficiency on the QTP. Carbohydrate metabolism provides energy through a series of complex chemical reactions, and lipid metabolism involves the fat breakdown and storage for energy. Of all PSGs, we identified two and nine PSGs that were associated with carbohydrate and lipid metabolism, respectively. Seven of these nine PSGs related to lipid metabolism were identified in a population comparison (Table S22, S25, and S29). The identified PSGs in those biological processes might reflect that *S. o'connori* underwent natural positive selection for energy metabolism and nutrient absorption to successfully adapt to ecological environments among populations.

### Conclusion

Formed by the collision between India and Eurasia, the QTP is the youngest, largest, and highest plateau on Earth. Due to its increasing uplift during the last 50 Ma, organisms endemic to the QTP are useful models for investigating the underlying molecular mechanisms for adaptations to rigorous high-land environments. Many fish species in the subfamily Schizothoracinae are polyploids, and scientists believed that WGD played an important role in the environmental adaptation and speciation of Schizothoracinae.

*S. o'connori* is thought to be a representative tetraploid species in the subfamily Schizothoracinae. We assembled a reference genome for this species and confirmed its genomic features as a tetraploid fish. As this species has undergone a recent WGD (∼1.23 mya), *S. o'connori* is an excellent model to study molecular mechanisms of early re-diploidization after WGD and environmental adaptations on the QTP. We demonstrated that transposon expansion can facilitate genome re-diploidization by prompting sequence mutagenesis and altering gene expression regulation, resulting in sequence divergence as well as function differentiations. In contrast to the tetraploid genome of *S. salar*, we did not observe massive inter-chromosomal rearrangements. We propose that the genome of *S. o'connori* represents an underway stage of re-diploidization for polyploidy after WGD and provides a golden opportunity to investigate post-WGD early genome evolution. To investigate putative functional genes contributing to the survival and environmental adaptation of *S. o'connori* to the harsh QTP environment, we identified PSGs through ortholog, ohnolog, and population comparisons and found that these genes were involved in DNA repair, folate transport and metabolism, energy metabolism, and nutrient absorption.

### Limitations of the Study

We confirmed genome features of the *S. o'connori* using reference genome sequences, but the molecular mechanism of WGD in *S. o'connori* was still not clear. The allotetraploid or autotetraploid nature of *S. o'connori* need further experiments. Meanwhile, we observed massive transposon expansion in the *S. o'connori* genome and found that insertions of transposon could induce the re-diploidization both on sequence and gene expression. However, we did not observe large-scale genomic rearrangements as in *S. salar* genome. Whether those genomic features are shared among endemic Schizothoracinae fish on the QTP need more genome investigations.

### Resource Availability

#### Lead Contact

Further information for the research should be directed to the Lead Contact Haiping Liu (luihappying@163.com).

#### Materials Availability

This study did not generate new unique reagents.

#### Data and Code Availability

Genome and transcriptome sequencing data were submitted to the National Center for Biotechnology Information (NCBI) BioProject number PRJNA557578. Genomic sequencing data from Pacbio SMRT, Hi-C, and Survey are available from the NCBI Short Read Archive as SRR10018089–SRR10018098, SRR10018101–SRR10018116, and SRR9964271–SRR9964278, respectively. To facilitate the re-production of our results and codes re-use, we have released our scripts in the github: https://github.com/DingDingFan/Whole-genome-duplication/tree/master/.

## Methods

All methods can be found in the accompanying Transparent Methods supplemental file.

## Supplemental Information

Document S1. Figures S1–S21 and Tables S1–S19, S21, S23, S24, S26–S28, and S30–S33

Table S20. Summary of RNA-Seq for *Danio rerio* in WGCNA, Related to Figure 4

Table S22. Positive Selection Genes Detected by Codeml with Branch Site Model from PAML (-v 4.9 g) in *S. o'connori* genome, Related to Figure 5 and Table 2

Table S25. Ohnolog Genes under Positive Selection (Ka/Ks > 1) Detected by KaKs_Calculator v2.0 in *S. o'connori* Genome, Related to Figure 5

Table S29. Summaries of the 306 Positively Selected Genes Detected by Whole Genome Sequencing Methods in the *Schizothorax o'connori*, Related to Figure 5
