# Supplementary material for: Genome of Tetraploid Fish Schizothorax o'connori Provides Insights into Early Re-diploidization and High-Altitude Adaptation
Source: iScience. 2020 Aug 22;23(9):101497. doi: 10.1016/j.isci.2020.101497 (PMC7486454; doi:10.1016/j.isci.2020.101497)
Supplement: Document S1. Figures S1–S21 and Tables S1–S19, S21, S23, S24, S26–S28, and S30–S33 [file mmc1.pdf]

iScience, Volume *23*

Supplemental Information

Genome of Tetraploid Fish *Schizothorax o'connori* Provides Insights into Early Re-diploidization and High-Altitude Adaptation

Shijun Xiao, Zhenbo Mou, Dingding Fan, He Zhou, Ming Zou, Yu Zou, Chaowei Zhou, Ruibin Yang, Jiaqi Liu, Shilin Zhu, Yajuan Li, Yanchao Liu, Fei Liu, Wanliang Wang, Benhe Zeng, Hong Li, Di Wang, and Haiping Liu

Supplementary Tables

Table S1 Short name and data resource used throughout the supplementary. Related to Figure 1.

Short name	Scientific name	Source	Note
SCHoco	*Schizothorax o'connori*	this study	this study
GLYmac	*Glyptosternon maculatum*	GigaScience	http://gigadb.org/dataset/100489
ICTpun	*Ictalurus punctatus*	Ensemble	release-94
ASTmex	*Astyanax mexicanus*	Ensemble	release-94
DANrer	*Danio rerio*	Ensemble	release-94
MEGamb	*Megalobrama amblycephala*	GigaScience	http://gigadb.org/dataset/100305
Ctransposonide	*Ctenopharyngodon idella*	-	http://www.ncgr.ac.cn/grasscarp
CARaur	*Carassius auratus*	NCBI	GCF_003368295.1
CYPcar	*Cyprinus carpio*	NCBI	GCF_000951615.1
SINgra	*Sinocyclocheilus grahami*	NCBI	GCF_001515645.1
SALsal	*Salmo salar*	NCBI	GCF_000233375.1

Table S2 High throughput data generated in this study and used for the assembly of the *Schizothorax o'connori* genome. The coverage was calculated using an estimated genome size of 1.94 Gb. Related to Figure 1 and Table 1.

Pair-end libraries	Insert size (bp)	Total data (Gb)	Read length (bp)	Sequence coverage (x)
Illumina reads	350	295.2	150	152.16
Pacbio reads	-	141	-	72.68
total	-	436.2	-	224.85

Table S3 Statistics of 31-mer analysis. The genome size of *Schizothorax o'connori* was estimated to be ~1,938.3 Mb. Related to Figure 1 and Table 1.

K-mer	K-mer number	K-mer depth	Genome size（Mb）	Revised Genome size (Mb)	Repeat (%)
31	209,524,858,560	99	2,116.41	1,938.30	56.65

Table S4 Summary of genome assembly of *Schizothorax o'connori*. Related to Figure 1 and Table 1.

	Contig length (bp)	Contig number
Total	2,067,143,420	25,126
N50	241,912	2,299
N60	190,413	3,265
N70	143,270	4,514
N80	100,040	6,236
N90	53,827	8,984

Table S5 Summary of RNA-seq for *Schizothorax o'connori*. Related to Figure 4.

Sample	Total reads	Total Bases (bp)	GC (%)	Q20 (%)	Q30 (%)
brain1	54,568,572	8,185,285,800	44.60	96.57	91.57
brain2	54,687,332	8,203,099,800	44.78	96.41	91.22
brain3	46,970,020	7,045,503,000	45.51	96.50	91.37
bladder1	50,779,150	7,616,872,500	46.97	96.06	90.45
bladder2	48,186,636	7,227,995,400	46.89	95.23	88.79
bladder3	52,519,810	7,877,971,500	47.04	96.32	90.97
blood1	43,376,542	6,506,481,300	45.7	96.32	91.02
blood2	44,359,962	6,653,994,300	47.27	96.52	91.31
blood3	58,230,356	8,734,553,400	46.71	96.47	91.28

eye1	51,750,466	7,762,569,900	46.2	96.61	91.61
eye2	57,056,460	8,558,469,000	46.18	96.31	91.00
eye3	53,709,888	8,056,483,200	46.11	96.35	91.09
fat1	59,750,130	8,962,519,500	50.19	96.97	92.28
fat2	46,084,572	6,912,685,800	49.55	96.4	91.22
fat3	41,815,914	6,272,387,100	51.42	95.75	89.42
gill1	52,421,886	7,863,282,900	45.78	96.55	91.52
gill2	49,374,968	7,406,245,200	45.92	96.68	91.72
gill3	44,221,190	6,633,178,500	46.03	96.22	90.82
heart1	58,951,096	8,842,664,400	45.86	96.75	91.93
heart2	46,783,092	7,017,463,800	46.00	96.47	91.25
heart3	46,769,590	7,015,438,500	46.16	96.20	90.76
intestine1	60,350,596	9,052,589,400	45.95	96.56	91.57
intestine2	56,615,052	8,492,257,800	46.61	96.65	91.66
intestine3	46,353,418	6,953,012,700	47.05	96.53	91.40
kidney1	50,731,352	7,609,702,800	45.90	96.38	91.16
kidney2	44,966,134	6,744,920,100	45.62	96.59	91.56
kidney3	51,202,892	7,680,433,800	45.90	96.35	91.08
liver1	67,776,260	10,166,439,000	46.41	96.84	92.06
liver2	50,256,982	7,538,547,300	46.22	96.34	90.98
liver3	51,840,710	7,776,106,500	46.67	96.39	91.09
mucle1	60,483,060	9,072,459,000	47.91	96.56	91.5

mucle2	48,829,500	7,324,425,000	47.52	96.56	91.48
mucle3	48,228,046	7,234,206,900	48.34	96.16	90.56
ovary1	59,078,620	8,861,793,000	48.12	94.97	88.56
ovary2	46,406,332	6,960,949,800	48.11	95.96	90.35
ovary3	44,446,606	6,666,990,900	47.90	95.90	90.30
skin1	42,995,272	6,449,290,800	47.19	96.32	91.00
skin2	48,854,244	7,328,136,600	47.32	96.06	90.43
skin3	60,968,926	9,145,338,900	46.50	96.66	91.73
spleen1	63,788,050	9,568,207,500	46.08	96.69	91.82
spleen2	51,046,618	7,656,992,700	46.18	96.31	90.99
spleen3	57,616,588	8,642,488,200	46.24	96.36	91.10
tentacles1	69,319,130	10,397,869,500	45.55	96.16	90.76
tentacles2	50,091,782	7,513,767,300	47.71	96.39	91.09
tentacles3	52,597,910	7,889,686,500	47.18	96.48	91.29

Table S6 Summaries of genes of *Schizothorax o'connori* and related species. ORF means open reading frame. Related to Figure 1 and Table 1.

**Species**	**Gene number**	**ORF (%)**	**Single exon (%)**	**Average transcript length (bp)**	**Average CDS length (bp)**	**Average exons per gene**	**Average exon length (bp)**	**Average intron length (bp)**
G. maculatum	22,846	94.73	6.65	17,782.28	1,762.04	10.31	170.85	1,720.14
A. mexicanus	23,042	67.88	5.53	20,457.70	1,588.71	9.81	161.97	2,142.10
D. rerio	25,591	84.74	7.20	25,474.69	1,613.27	9.33	172.86	2,863.59
C. idellus	32,691	59.86	10.44	10,400.72	1,387.00	7.66	181.02	1,353.01
S. grahami	45,778	91.33	6.30	16,259.73	1,587.47	9.25	171.70	1,779.42
C. carpio	48,287	91.46	5.45	10,990.49	1,266.81	7.71	164.22	1,448.24
S. salar	48,334	85.31	6.71	89,751.94	1,568.95	8.68	180.86	11,706.71
C. auratus	52,870	98.22	7.11	14,029.56	1,709.58	9.67	176.86	1,421.59
S. o'connori	43,731	83.61	7.94	13,389.89	1,508.89	8.85	170.49	1,513.45

Table S7 Summaries of BUSCO assessments for the gene annotation of *Schizothorax o'connori* and related species. Related to Figure 1 and Table 1.

Species	Complete (%)	Complete single-copy (%)	Complete duplicated (%)	Fragmented (%)	Missing (%)
G. maculatum	98.3	94.8	3.5	0.7	1.0
S. salar	98.1	47.5	50.6	1.2	0.7
D. rerio	97.0	77.0	20.0	1.6	1.4
A. mexicanus	93.0	88.7	4.3	5.1	1.9
C. idellus	85.4	81.6	3.8	11.6	3.0
C. carpio	83.8	47.3	36.5	11.7	4.5
S. o'connori	92.1	40.9	51.2	4.3	3.6
C. auratus	99.3	30.2	69.1	0.3	0.4

Table S8 Summaries of functional annotations for *Schizothorax o'connori*. Related to Figure 1 and Table 1.

**Database**		**Annotated Number**	**Annotated Percent(%)**
**NR**		43,400	99.24
**Swiss-Prot**		40,909	93.55
**KEGG**		37,442	85.62
**InterPro**	**All**	43,675	99.87
	**Pfam**	38,337	87.67
	**GO**	41,331	94.51
**Annotated**		43,719	99.97
**Total**		43,731	-

Table S9 Summary of RBH (reciprocal best hit) orthologs and Ohnologues for each species. All orthorlogs should have an identity >60%, coverage > 50% and alignment score >200. For ohnologues, the identity differences between the first and the second-best hit were less than 10%. Related to Figure 1 and Table 1.

Species	Orthology	Orthology (%)	Orthology of zebrafish Gene	Orthology of zebrafish gene (%)	Ohnolog	Ohnolog (%)	Total genes
G．maculatum	12,009	47.93	12,009	47.15	-	-	25,054
C． auratus	51,105	96.65	20,619	80.95	28,264	53.45	52,875
C． idellus	17,240	52.74	17,240	67.68	-	-	32,691
C． carpio	46,431	96.16	19,995	78.50	22,130	45.83	48,287
M．amblycephala	16,839	71.06	16,839	66.11	-	-	23,696
S． o'connori	42,348	96.84	19,103	75.00	25,670	58.70	43,731
S．grahami	44,912	98.11	20,407	80.12	31,010	67.74	45,778

Table S10 Summary of homoeologous regions resulted from whole genome duplication. Related to Figure 1 and Table 1.

Classification	Bases (bp)	Number	N50 (bp)
R1	834,176,523	5,379	256,341
R2	927,726,323	4,237	298,351
Unclass	305,240,574	15,510	55,679

Table S11 Genomes selected for gene family constructions in the *Schizothorax o'connori* genome project. Related to Figure 1.

Species	order	Rounds of WGD
D. rerio	Cypriniformes	3
C. idellus	Cypriniformes	3
M. amblycephala	Cypriniformes	3
C. carpio	Cypriniformes	4
C. auratus	Cypriniformes	4
S. grahami	Cypriniformes	4
S. o'connori	Cypriniformes	4
G. maculatum	Siluriformes	3

Table S12 Summary of gene family in *Schizothorax o'connori* and related species. Related to Figure 1.

Species	Total genes	Unclustered genes	Families	Unique families	Average genes per family
D. rerio	25,471	486	16,160	48	1.55
M. amblycephala	23,696	354	15,460	27	1.51
C. idella	32,691	5,406	17,738	239	1.54
C. auratus	52,875	557	17,575	57	2.98
C. carpio	48,287	3,124	19,107	446	2.36
S. grahami	45,778	1,316	18,384	89	2.42
S. o'connori	43,731	385	16,576	101	2.61
G. maculatum	25,054	5,382	17,976	441	1.09

Table S13 Divergence time estimation based on the Ks values of orthologs (ohnologs) between (sub)genomes. On the basis of a Ks rate of 3.51×10^{-9} substitutions per synonymous site per year and the obtained Ks value of 0.0086 of *S. o'connori* , we estimated that the latest WGD (4R) happened 1.23 million years ago. The Confidence Intervals were calculated using t.test function in R program with 95% confidence level. For *Salmo salar* we cited from previous study. Related to Figure 2.

**Species**	**Ks peak**	**Divergence time (Ma)**	**Confidence Intervals**	
S. o'connori	0.0086	1.23	1.15	1.31
C. auratus	0.1869	26.62	26.47	26.78
C. carpio	0.1405	20.02	19.82	20.22
S. grahami	0.1202	17.12	17.02	17.24
S. salar	0.2187	80	--	--

Table S14 Summaries of transposable element (transposon) annotations for *Schizothorax o'connori*. Related to Figure 2.

**Method**	**Repeat Size**	**% of genome**
Repeatmasker	533,537,803	25.81
RepeatModeler	903,565,528	43.71
RepeatScout	856,195,906	41.42
Total	1,039,571,554	50.29

Table S15 Summaries of transposon classifications according to each method for *Schizothorax o'connori*. Related to Figure 2.

Type	Repbase transposons		***De novo***		Combined transposons	
	**Length (bp)**	**% in genome**	**Length (bp)**	**% in genome**	**Length (bp)**	**% in genome**
DNA	341,560,285	16.52	605,805,104	29.31	641,238,766	31.02
LINE	116,765,649	5.65	147,181,163	7.12	157,195,043	7.60
SINE	9,223,933	0.45	14,065,750	0.68	14,307,778	0.69
LTR	56,239,694	2.72	79,008,023	3.82	88,886,613	4.30
Other	3,125	0.00	0	0.00	2,710	0.00
Unknown	0	0.00	132,197,018	6.40	132,197,018	6.40
Total	519,069,683	25.11	954,550,195	46.18	1,000,999,455	48.42

Table S16 Summary of transposons content in 14 genomes from Repbase (2018.12) and repeatScout result. The species with background orange color means the genome was tetraploid genome. Related to Figure 2.

Order	Species	DNA	LINE	LTR	SINE	Unknown	Total
Salmoniformes	*S. salar*	23.08	12.08	5.72	1.20	2.97	46.61
Salmoniformes	*O. mykiss*	24.91	13.19	5.09	1.61	4.65	48.36
Cypriniformes	*D. rerio*	45.87	4.42	5.94	1.67	0.56	57.61
Cypriniformes	*C. idella*	26.21	1.33	2.20	0.18	5.50	35.55
Cypriniformes	*M. amblycephala*	28.35	2.26	4.45	0.19	9.66	45.05
Cypriniformes	*S. grahami*	21.69	2.74	4.02	0.36	5.71	35.26
Cypriniformes	*C. auratus*	22.40	5.71	5.56	0.85	4.42	38.98
Cypriniformes	*C. carpio*	20.14	4.37	3.63	0.60	5.73	34.99
Cypriniformes	*S. o'connori*	28.04	7.36	4.21	0.62	3.15	43.17

Table S17 transposon family great than 1% bases (filled yellow cells) in *Schizothorax o'connori*genome from repeatScout result. The result was used to calculate the transposon burst time. The Species with background orange color highlighted tetraploid genome. Related to Figure 2.

**Type**	***O. mykiss***	***S. salar***	***D. rerio***	***C. idella***	***M. amblycephala***	***S. o'connori***	***C. carpio***	***C. auratus***	***S. grahami***
DNA/CMC	0.54	0.13	5.31	1.92	2.73	1.36	1.42	1.69	1.62
DNA/Crypton	0.39	0.19	1.19	0.45	0.36	0.56	0.32	0.74	0.35
DNA/hAT	3.83	2.78	11.25	3.76	4.58	6.17	4.17	5.00	5.50
DNA/Helitron	0.02	0.01	2.57	0.76	0.98	0.85	0.74	1.35	0.41
DNA/Kolobok	0.04	0.02	3.33	5.99	6.62	3.41	3.29	2.81	4.10
DNA/MULE	0.00	0.00	0.23	0.13	0.21	1.28	0.09	0.22	0.05
DNA/PIF	0.34	0.18	4.11	1.50	1.86	1.54	0.90	1.21	1.19
DNA/TcMar	14.29	11.17	4.66	2.99	2.95	6.01	3.04	2.82	2.42
LINE/L1	1.02	0.71	0.74	0.01	0.15	1.89	0.31	0.88	0.18
LINE/L2	5.07	5.10	1.61	0.60	0.93	2.52	2.12	3.04	1.35
LINE/Rex-Babar	4.64	3.62	0.33	0.04	0.09	1.85	0.89	0.76	0.38
LINE/Rtransposon	1.35	1.35	0.23	0.00	0.08	0.08	0.08	0.18	0.04
LTR/Copia	0.97	1.47	0.00	0.00	0.00	0.01	0.00	0.01	0.00
LTR/DIRS	0.05	0.04	1.05	0.68	1.14	0.87	1.08	1.28	1.57
LTR/ERV1	0.50	0.64	0.76	0.10	0.37	0.49	0.50	0.74	0.23
LTR/Gypsy	2.34	2.03	1.61	0.39	1.27	1.41	1.07	2.22	1.10
SINE/MIR	0.00	0.00	0.00	0.00	0.00	0.00	0.01	0.01	0.00
SINE/tRNA	1.08	0.74	0.04	0.00	0.05	0.14	0.18	0.09	0.06
SINE/tRNA-V	0.00	0.00	0.71	0.00	0.00	0.00	0.00	0.00	0.00

Table S18 HSP similarity detected by LASTZ self-genome aligment for *Schizothorax o'connori*and *Salmo salar*. Related to Figure 3.

>=Similarity(%)	*S. o'connori*			*S. salar*		
	HSP	Bases	% (Aligned)	HSP	Bases	% (Aligned)
20	2,546,344	785,064,602	100.00%	7,087,842	1,162,403,509	100.00%
50	2,541,414	784,753,816	99.96%	7,018,452	1,159,904,473	99.79%
60	2,431,560	772,967,794	98.46%	6,425,960	1,119,035,522	96.27%
70	2,256,276	755,186,316	96.19%	4,735,508	955,544,663	82.20%
90	1,865,654	717,796,843	91.43%	793,106	282,555,870	24.31%
95	1,595,226	671,310,201	85.51%	392,872	175,177,305	15.07%
96	1,495,270	645,611,039	82.24%	331,564	157,259,638	13.53%
97	1,354,402	603,855,740	76.92%	267,196	139,908,672	12.04%
98	1,143,210	526,095,462	67.01%	206,324	121,209,138	10.43%
99	772,774	343,972,720	43.81%	144,814	96,745,885	8.32%
100	278,296	47,559,101	6.06%	64,936	17,818,418	1.53%

Table S19 Insertion and deletion variants identification for the R1 and R2. Indel means insert and deletion between in R1 and R2. SNP means substitution between WGD fragments. Small indel means deletion/insertions in WGD aligned blocks ranged from 1 to 159 bp. Related to Figure 3.

Variation Type	Num	Bases	Percentage	Average happen size (bp)
Indel	271,251	179,996,077	8.71%	7,621
SNP	10,628,445	10,628,445	0.51%	165
Small indel	5,385,400	18,275,005	0.88%	338
Total	--	208,899,527	10.11%	--

Table S21 KEGG pathway enrichment analysis with species-specific in *Schizothorax o'connori* gill expression module. The enrichment was made by David 6.8. Related to Figure 4.

Term	Count	P-value	FDR
Cytokine-cytokine receptor interaction	18	1.37×10^{-4}	0.153829
Proteasome	10	6.91×10^{-4}	0.774936
Adherens junction	12	0.005101	5.595715
Tight junction	12	0.00968	10.37445
Endocytosis	23	0.018771	19.2141
Cell adhesion molecules (CAMs)	13	0.019247	19.65448
Jak-STAT signaling pathway	11	0.026025	25.68969
Mucin type O-Glycan biosynthesis	5	0.031393	30.17304
VEGF signaling pathway	9	0.032722	31.24428
Protein processing in endoplasmic reticulum	15	0.033383	31.77132
Apoptosis	8	0.051323	44.74722
ECM-receptor interaction	8	0.071888	56.83032
TGF-beta signaling pathway	9	0.077451	59.65602

Table S23 Gene ontology enrichment of genes for natural positively selected genes. Stars in the table represented that corresponding GO terms were significantly enrichmed among natural positively selected genes identified by various methods. Related to Figure 5.

GO term	ortholog	ohnolog	population
cell-cell adhesion	*	*	
mannosyl-oligosaccharide 1,2-alpha-mannosidase activity	*		
phospholipid catabolic process"	*	*	
phospholipase activity	*	*	
mismatched DNA binding	*	*	
intermediate filament cytoskeleton organization	*		*
intermediate filament	*		*
troponin complex	*		*
integrin complex	*		*
MHC class II protein complex	*		*
transferase activity transferring hexosyl groups	*		*
antigen processing and presentation	*		*
keratin filament	*		
alpha-mannosidase activity	*		
mannose metabolic process	*		
proton transport	*		
protein-L-isoaspartate (D-aspartate) O-methyltransferase activity	*		
actin cytoskeleton reorganization	*		
transferase activity transferring glycosyl groups	*		
microtubule organizing center	*		
phosphoric diester hydrolase activity	*		
glycerophosphodiester phosphodiesterase activity	*		
DNA repair	*		
cellular response to DNA damage stimulus	*		
immune response	*		

structural molecule activity	*		
nucleosome assembly		*	
chemokine activity		*	
protein dimerization activity		*	
multicellular organism development		*	
receptor binding		*	
mismatch repair		*	
DNA integration			*
voltage-gated potassium channel complex			*
nuclear envelope			*
guanylate cyclase activity			*
insulin-like growth factor binding			*
ion channel activity			*
ubiquitin-protein transferase activity			*
sensory perception of sound			*
lipoprotein metabolic process			*
neuron projection development			*
cGMP biosynthetic process			*

Table S24 KEGG biological pathway enrichment of genes for natural positively selected genes that identified from orthologs, ohnologs and population method. Stars in the table represented that corresponding KEGG pathways were significantly enrichmed among natural positively selected genes identified by various methods. Related to Figure 5.

KEGG pathway	ortholog	ohnolog	population
Cell adhesion molecules (CAMs)	*	*	*
Intestinal immune network for IgA production	*	*	*
Antigen processing and presentation	*	*	*
Linoleic acid metabolism	*	*	
Arachidonic acid metabolism	*	*	
Th1 and Th2 cell differentiation	*	*	
Apoptosis - multiple species	*	*	
Leukocyte transendothelial migration	*	*	
alpha-Linolenic acid metabolism	*	*	
NF-kappa B signaling pathway	*	*	
p53 signaling pathway	*	*	
Fanconi anemia pathway	*	*	
Hematopoietic cell lineage	*		*
Phagosome	*		*
Necroptosis	*		
MAPK signaling pathway - fly	*		
Collecting duct acid secretion	*		
Pentose and glucuronate interconversions		*	
Ether lipid metabolism		*	
Cytokine-cytokine receptor interaction		*	
Porphyrin and chlorophyll metabolism		*	
Osteoclast differentiation		*	
Homologous recombination		*	

Jak-STAT signaling pathway	*	
Protein export	*	
Steroid hormone biosynthesis	*	
Metabolism of xenobiotics by cytochrome P450	*	
Ferroptosis	*	
Serotonergic synapse		*
Fatty acid biosynthesis		*
Glycosphingolipid biosynthesis - globo and isoglobo series		*
Mucin type O-glycan biosynthesis		*
Glycosphingolipid biosynthesis - lacto and neolacto series		*
Renin secretion		*
mRNA surveillance pathway		*

Table S26 Summary of genome resequencing for the *Schizothorax o'connori* genome. Related to Figure 5.

Sample	Raw Base (bp)	Clean Base (bp)	Q20 (%)	Q30 (%)	GC Content (%)
Soco4700	100,884,532,500	100,555,179,600	97.00	95.46	38.80
Soco4000	99,544,687,800	99,066,715,500	96.77	94.86	38.38
Soco3100	113,226,539,400	112,599,624,300	96.88	95.26	38.59
Soco3600	109,571,165,700	109,310,314,800	97.02	95.56	38.39
Soco4200	97,375,310,700	97,218,252,300	96.93	95.33	38.42

Table S27 Mapping statistics summary of DNA resequencing clean data to the *Schizothorax o'connori* assembly. Related to Figure 5.

Sample	Total reads	Mapped reads	Mapping rate (%)	Average depth (X)	Coverage at least 1X (%)	Coverage at least 4X (%)
**Soco4700**	670,367,864	659,679,714	98.41	41.50	96.02	92.00
**Soco4000**	660,444,770	650,241,721	98.46	41.67	96.05	92.14
**Soco3100**	750,664,162	737,648,959	98.27	46.44	96.35	92.80
**Soco3600**	728,735,432	716,949,522	98.38	44.79	96.21	92.59
**Soco4200**	648,121,682	635,996,294	98.13	39.84	96.11	92.08

Table S28 Summaries of SNP annotated by ANNOVAR program for the *Schizothorax o'connori*. Related to Figure 5.

Sample	Exonic	Upstream	Downstream	Intergenic	Het rate(‰)	Total
**Soco4700**	181,403	201,721	192,191	5,338,764	3.88	8,608,643
**Soco4200**	180,202	201,451	191,563	5,315,069	3.88	8,567,019
**Soco4000**	180,618	202,510	193,237	5,365,074	3.91	8,649,913
**Soco3600**	180,934	205,314	194,417	5,401,142	3.95	8,702,258
**Soco3100**	183,343	206,028	195,309	5,452,834	3.97	8,775,788

Table S30 Statistics of variants calling. The short sequence reads generated by Illumina platform were mapped to the *Schizothorax o'connori* genome with BWA and performed variant calling with SAMtools to get the SNP result. Related to Figure 5.

	Number	Percentage (%)
All SNP	3,980,508	0.24
Heterozygosis SNP	3,911,059	0.24
Homology SNP	69,449	0.01

Table S31 ***Schizothorax o'connori* assembly completeness evaluated using CEGMA.** Related to Figure 1.

Species	Complete		Complete + Partial	
	**# Prots**	**%completeness**	**# Prots**	**%completeness**
S. o'connori	233	93.95	242	97.58

Table S32 Shared transposon identification in the *Schizothorax o'connori* and *Carassius auratus* genome. Related to Figure 2.

Species	Ratio of shared transposon length to whole genome length	Ratio of shared transposon length to total transposon length
S. o'connori	0.22	0.53
C. auratus	0.08	0.25

Table S33 Summary of gene lost in homologous regions of *Schizothorax o'connori*. Related to Figure 2.

	C. auratus	*S. grahami*	*S. o'connori*	*S. salar*
**Subgenome divergence time (Ma)**	26.62	17.12	1.23	80.00
**Synteny pair**	21,593	14,750	14,882	20,476
**Genes of synteny**	30,065	24,885	22,940	27,176
**Synteny Genes On same sequence**	1,872	0	542	565
**Multiple pair lost**	531	253	267	267
**Singleton**	2,246	2,695	1,546	999
**Singleton (%)**	11.70	18.59	10.99	5.09
**Singleton/Ma**	84	157	1,257	12
**Exonerate+tblastn**	1,074	1,300	760	470
**Gene lost**	1,172	1,395	786	529
**Lost/Ma**	44	81	639	7

Supplementary Figures

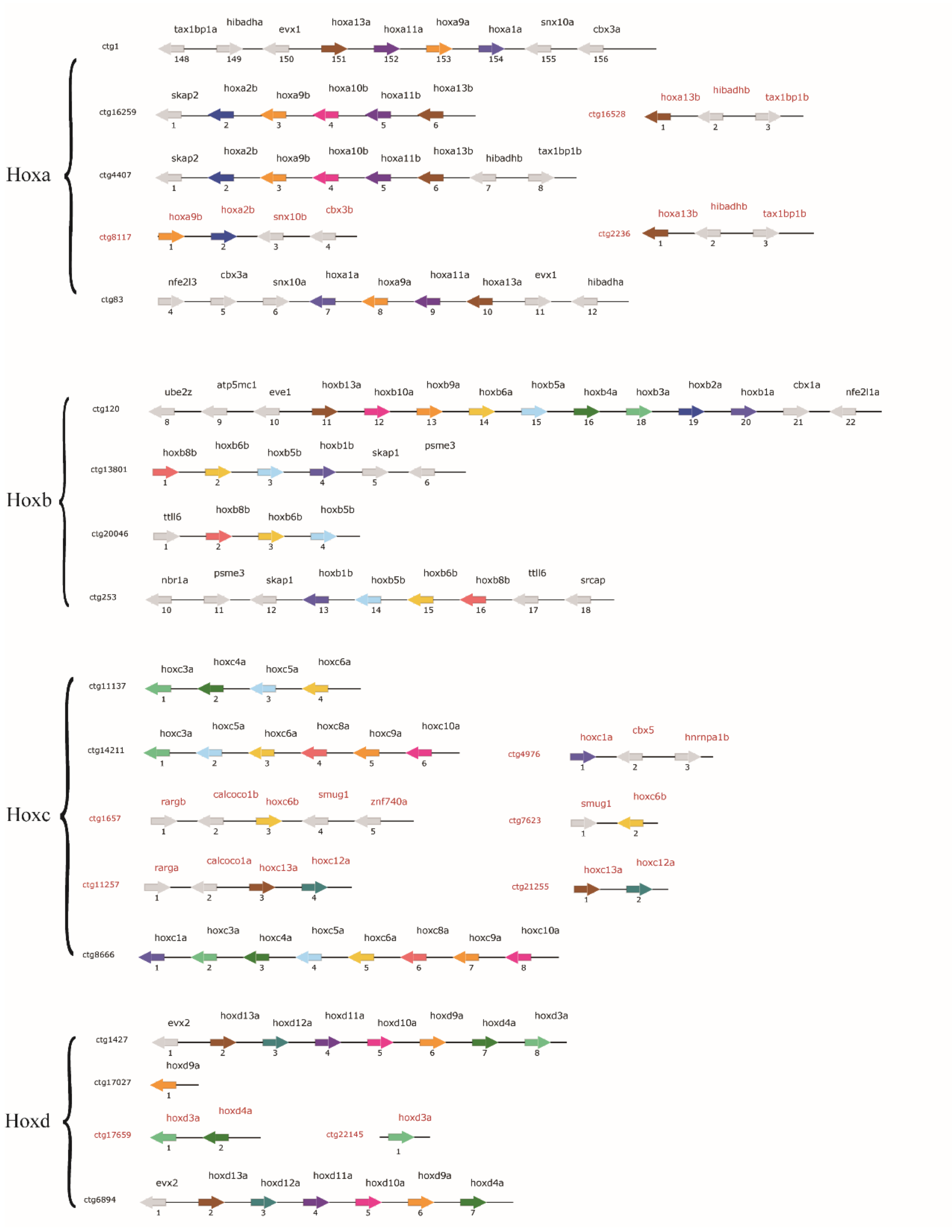

Figure S1 Hox gene clusters in *Schizothorax o'connori* genome. Related to Figure 1.

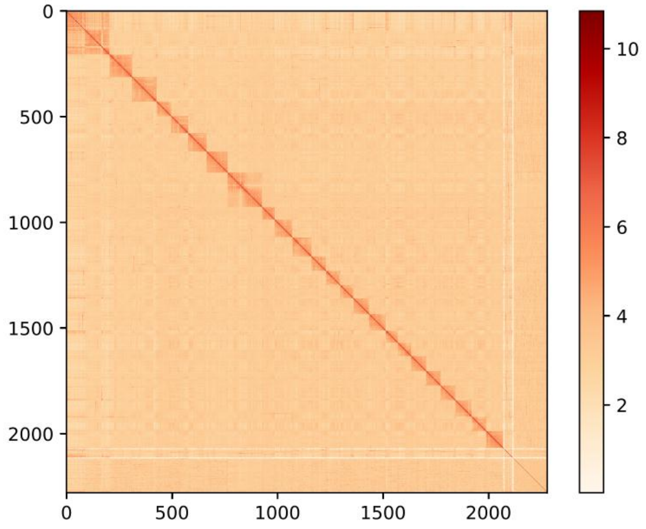

Figure S2 The whole genome Hic interaction map generated using a sliding window of 500 kb. Related to Figure 1.

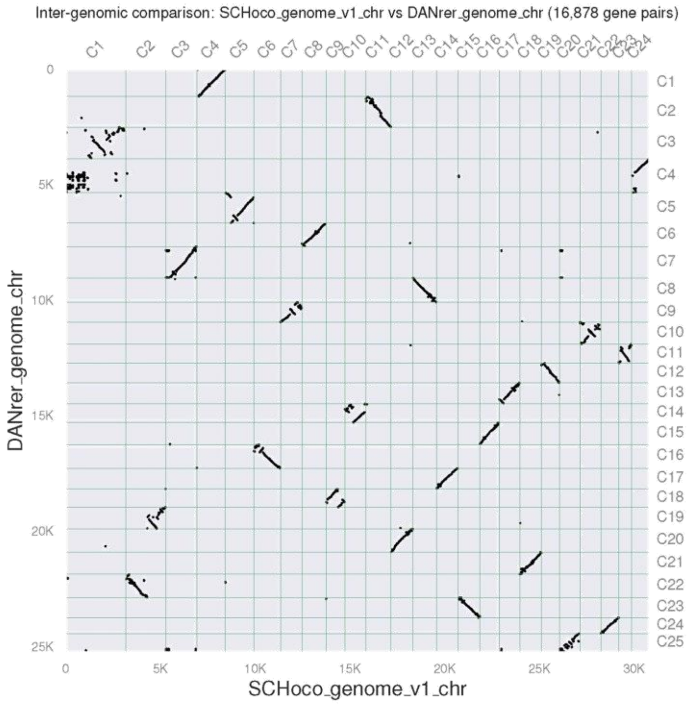

Figure S3. The whole-genome conservation synteny of *Schizothorax o'connori* and *Danio rerio*. Related to Figure 1.

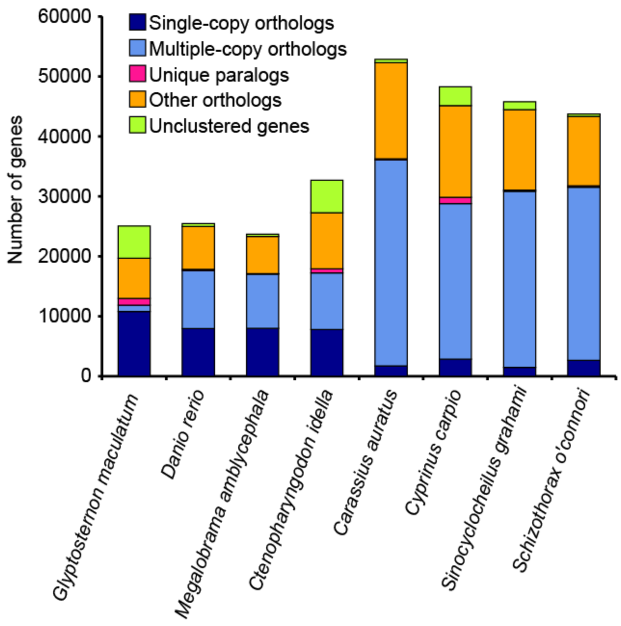

Figure S4 The orthologous relationships of *Schizothorax o'connori* and related species. Single-copy orthologs means that gene families have copy numbers of 1 in 8 species; Multiple-copy orthologs means gene families with copy numbers larger than 1 in 8 species; Unique paralogs means gene families with no homologous genes in other 7 species, but existed in one species; Other orthologs means gene families presented in the several species, but not existed in all 8 species. Unclustered genes means gene families that genes were not clustered together. Related to Figure 2.

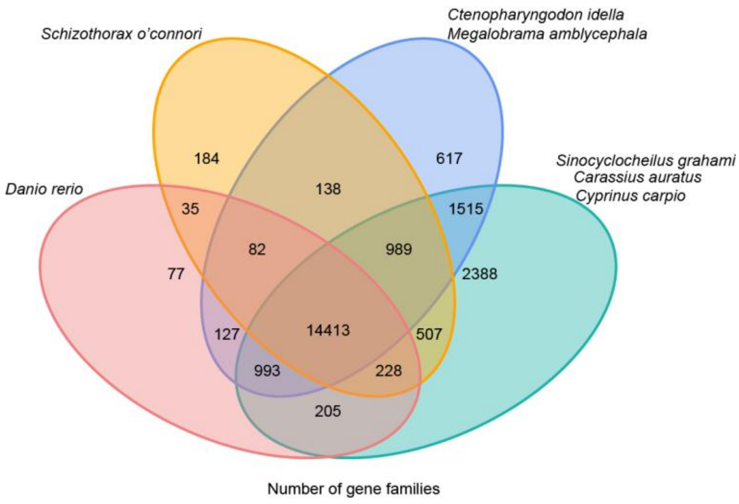

Figure S5 Venn diagram of the shared gene families in *Schizothorax o'connori* and related species. Related to Figure 2.

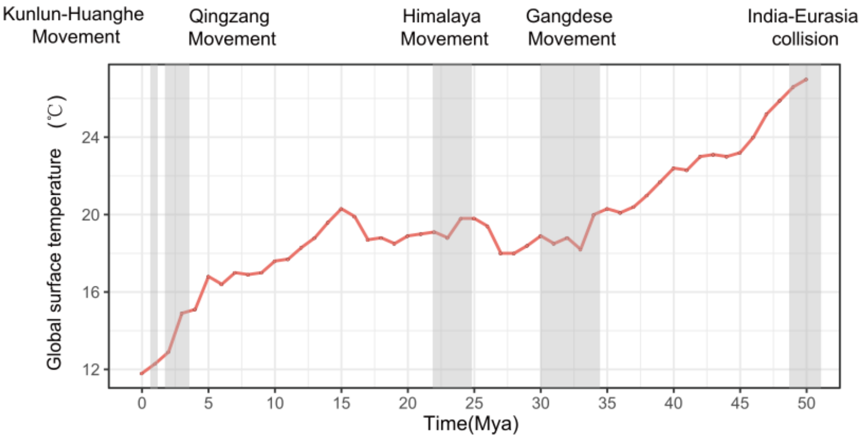

Figure S6 the dramatic tectonic movements in the Qinghai-Tibet Plateau and global surface temperature fluctuations after the India-Eurasia collision, shown by the gray bars. Related to Figure 2.

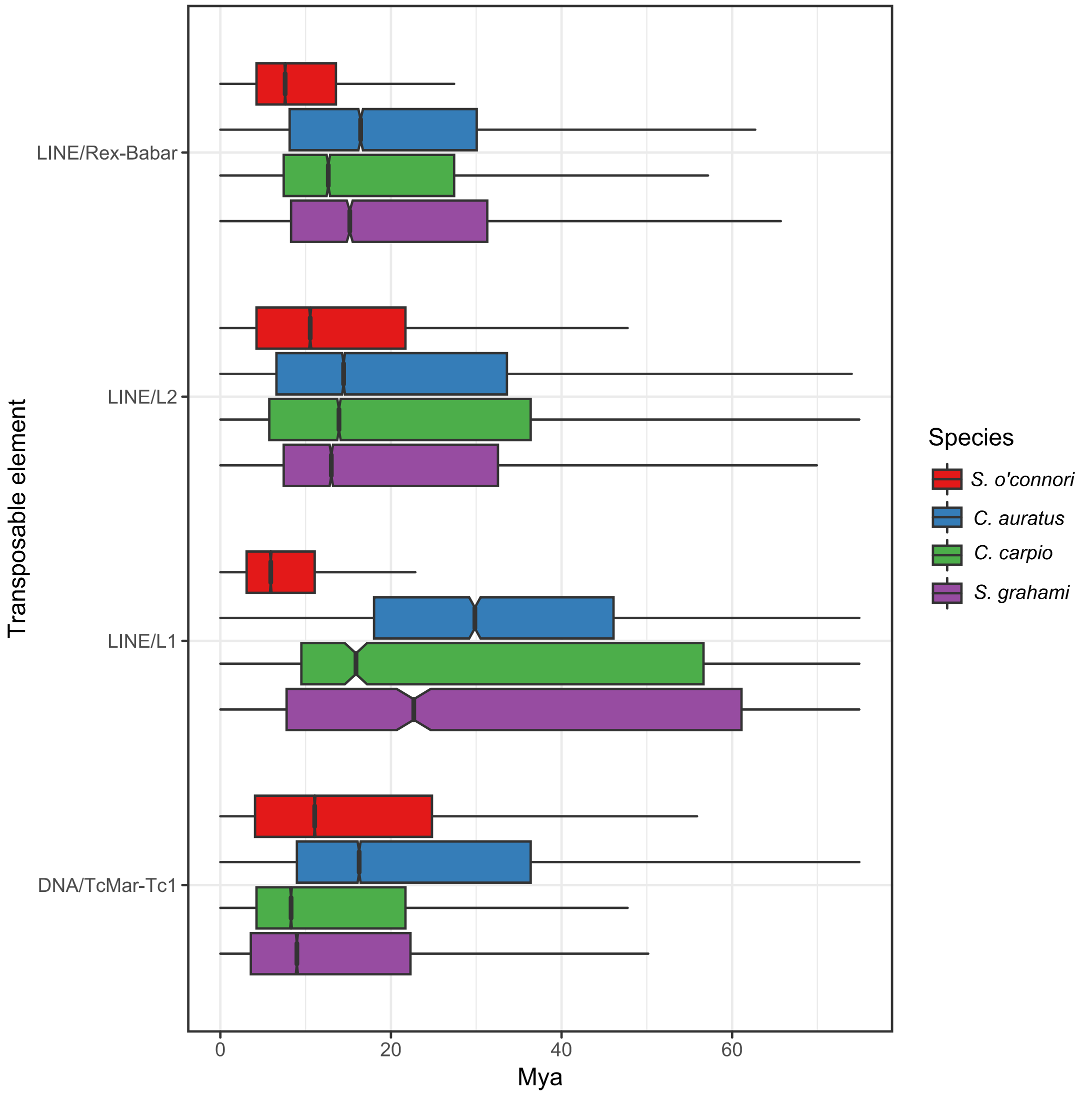

Figure S7 Boxplot display the Transposable elements divergence time calculated by using the synonymous substitution site per million years estimated for cyprinids (0.003121781). Related to Figure 2.

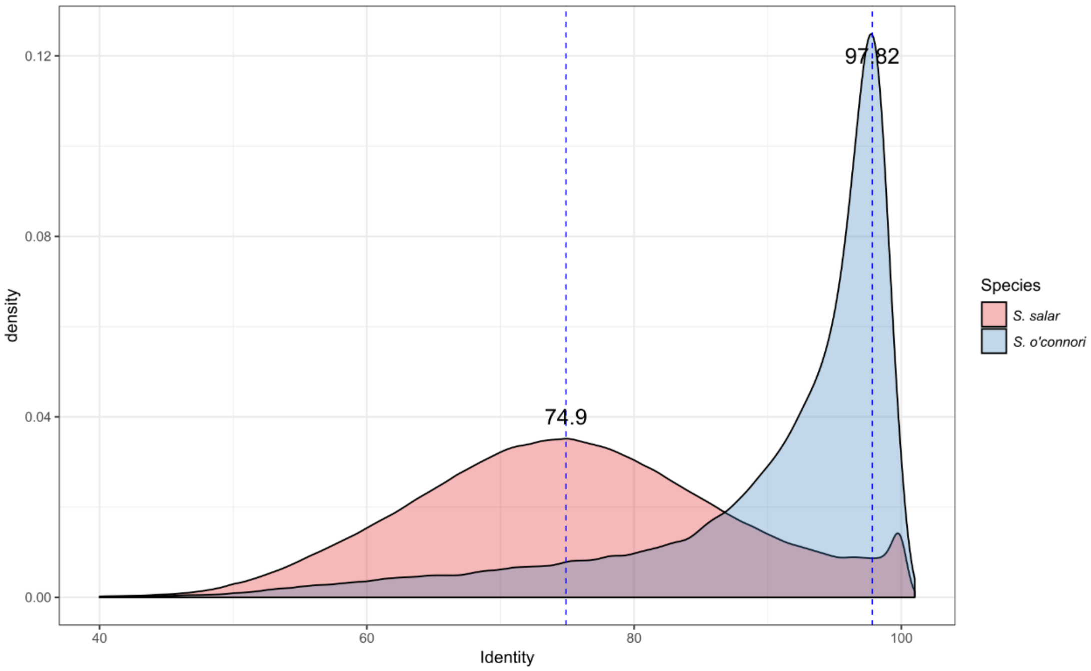

Figure S8 High score pair (HSP) similarity distribution for *Schizothorax o'connori* and Salmon homologous regions. Related to Figure 2.

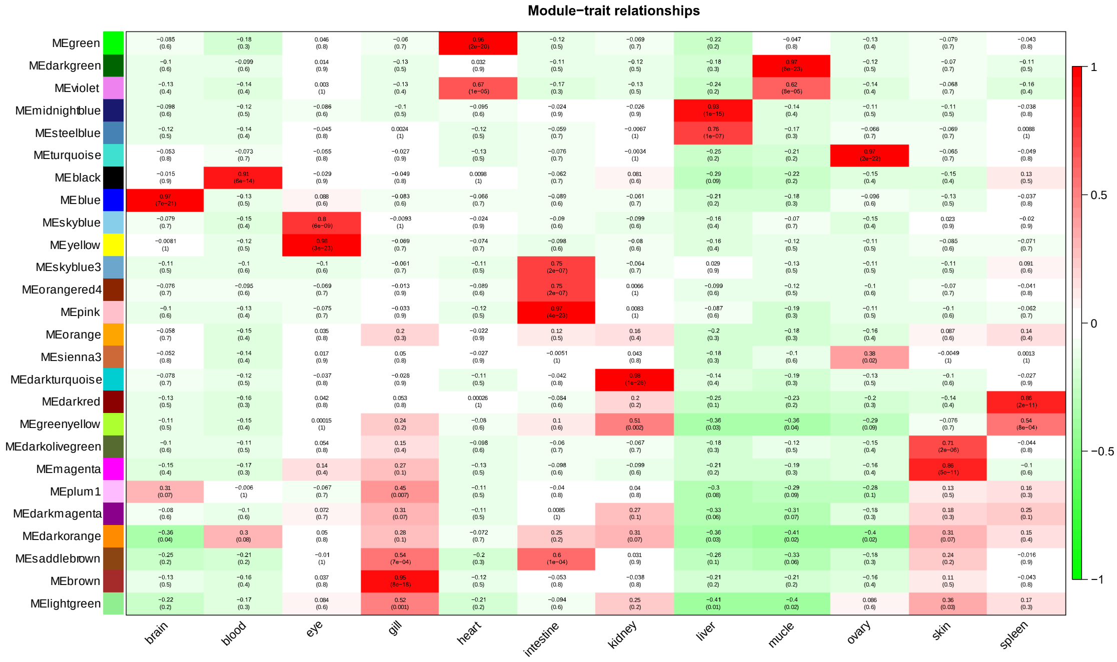

Figure S9 Model and tissue correlation and p-value for *Schizothorax o'connori* from WGCNA analysis. In each cell, the top value means Pearson correlation value and bottom value means p-value. Related to Figure 4.

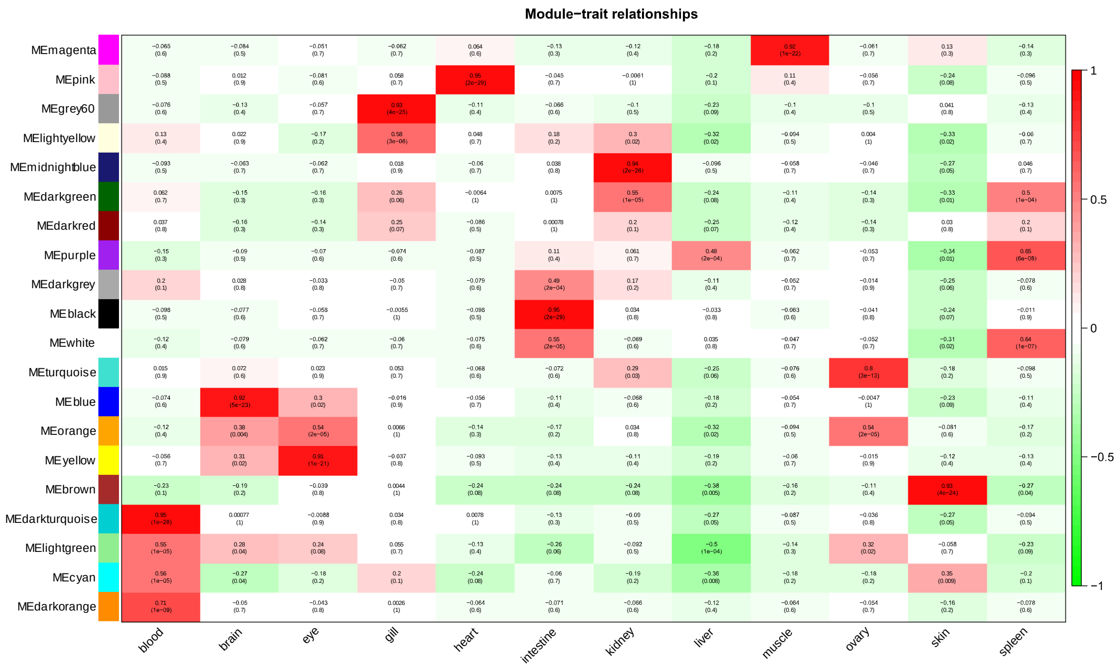

Figure S10 Model and tissue correlation and p-value for *Danio rerio* from WGCNA analysis. In each cell, the top value means Pearson correlation value and bottom value means p-value. Related to Figure 4.

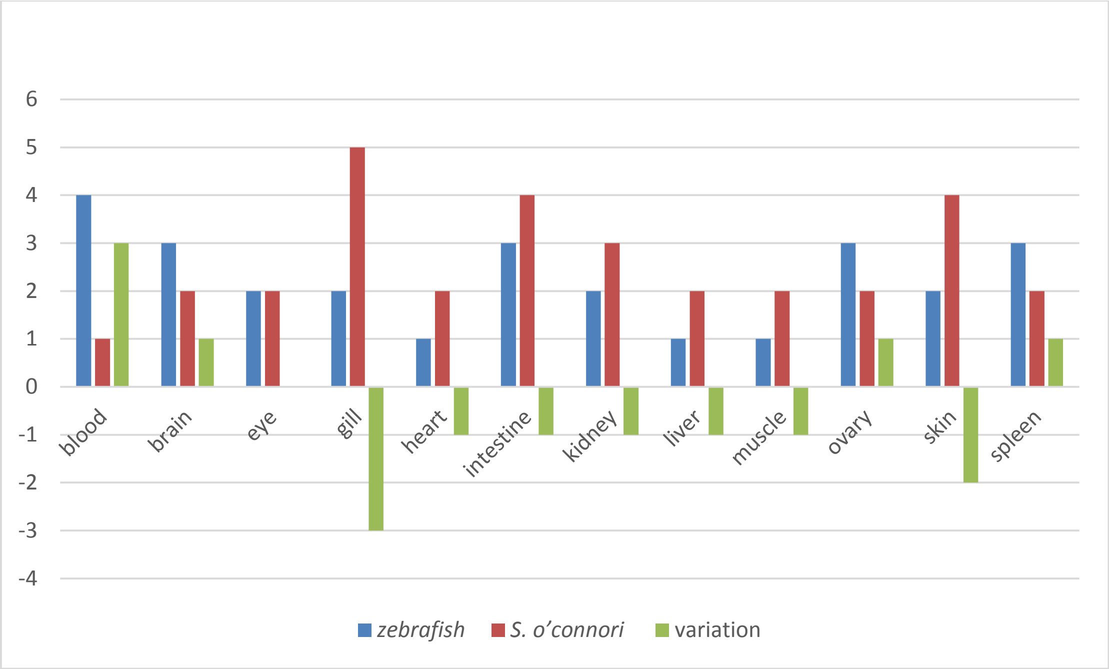

Figure S11 Model number for each tissue with correlation great than 0.3. Variation means the model number of *Danio rerio* minus that of *Schizothorax o'connori*. Related to Figure 4.

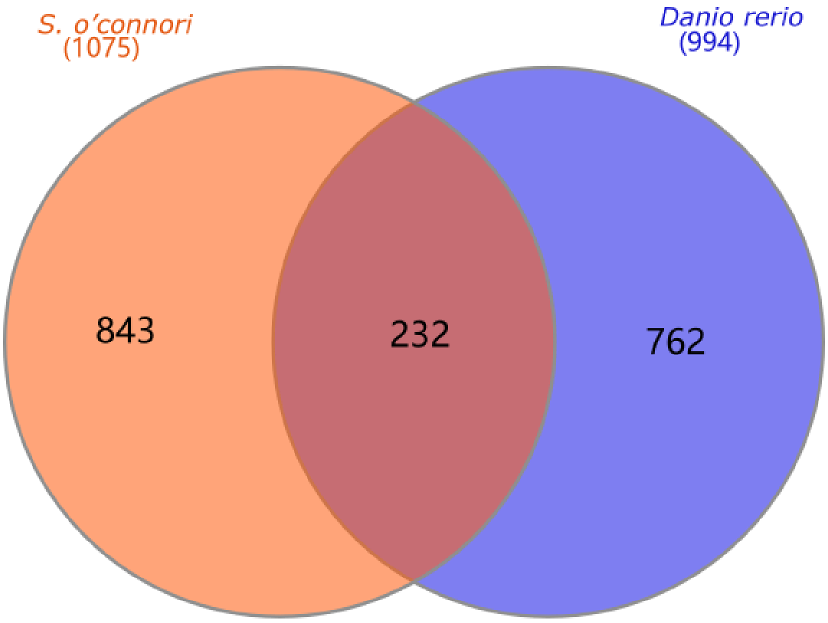

Figure S12 The Venn plot display the share genes in the top correlation modules of gill. We chose 1,699 *Schizothorax o'connori* homology genes (single best hit) to *D. rerio* to make the plot. Related to Figure 4.

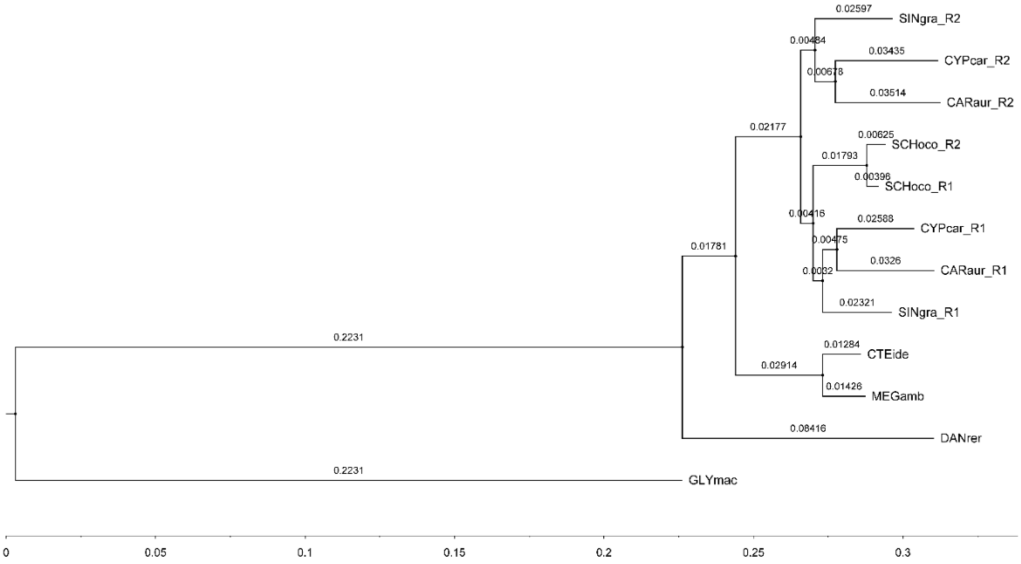

Figure S13 Phylogeny of the (sub)genomes of 8 species based on 297 genes totaling 566,916 nucleic acid sites. It is reconstructed using RAxML (-v 8.2.12) with GTRGAMMA model and 1,000 fast bootstrap replicates were also performed. The name of species were listed in Table S1. Related to Figure 2.

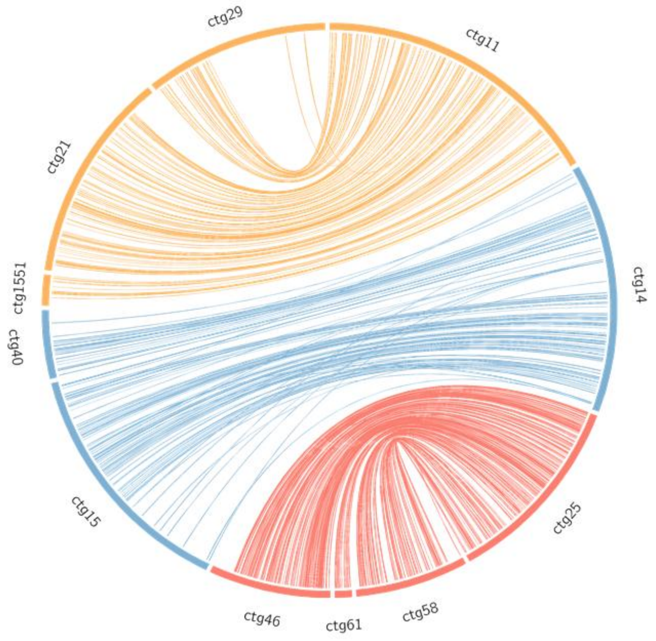

Figure S14. The synteny of shared transposon between duplicated genome regions for *Schizothorax o'connori*. Related to Figure 2.

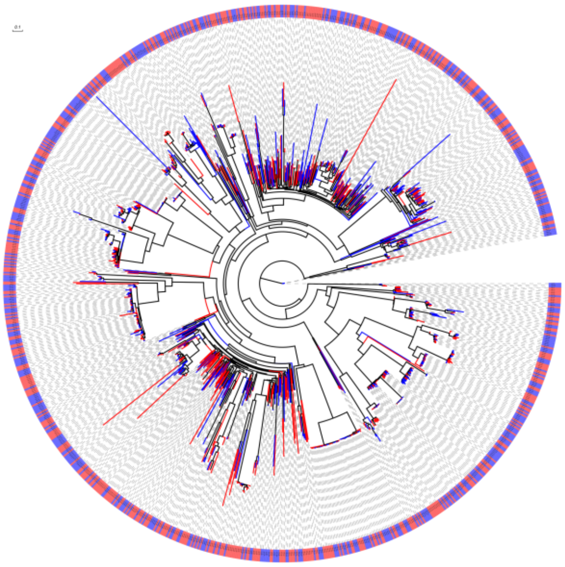

Figure S15. The phylogeny of 1,417 DNA/TcMar-Tc1 of ctg1 and reciprocal sequence in the *Schizothorax o'connori* genome. The red color represented the transposon from R1 and blue for R2. TEs with length <200bp were removed and the topology were inferred with Raxml (f a -m GTRGAMMA -# 100). Related to Figure 2.

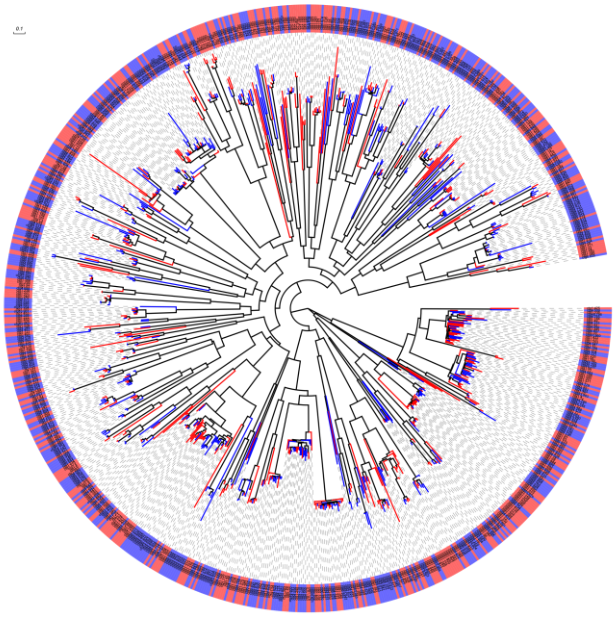

Figure S16. The phylogeny of 992 DNA/hAT-Ac of ctg1 and reciprocal sequence in the *Schizothorax o'connori* genome. The red color represented the transposon from R1 and blue for R2. TEs with length <200 bp were removed and the topology were inferred with Raxml (f a -m GTRGAMMA -# 100). Related to Figure 2.

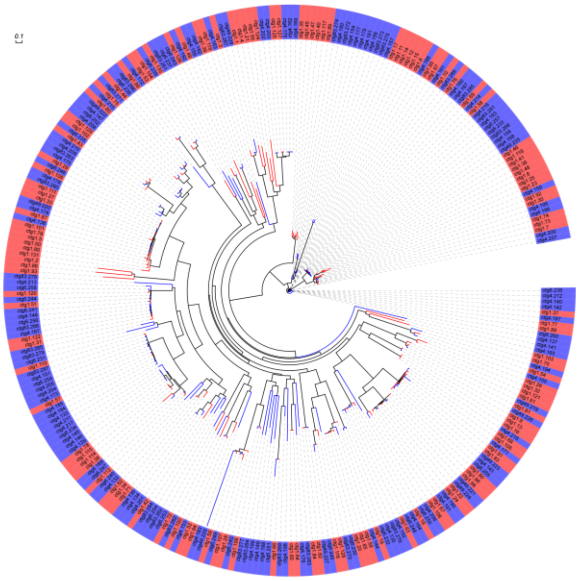

Figure S17. The phylogeny of 285 LINE/L2 of ctg1 and reciprocal sequence in the *Schizothorax o'connori* genome. The red color represented the transposon from R1 and blue for R2. TEs with length < 200 bp were removed and the topology were inferred with Raxml (f a -m GTRGAMMA -# 100). Related to Figure 2.

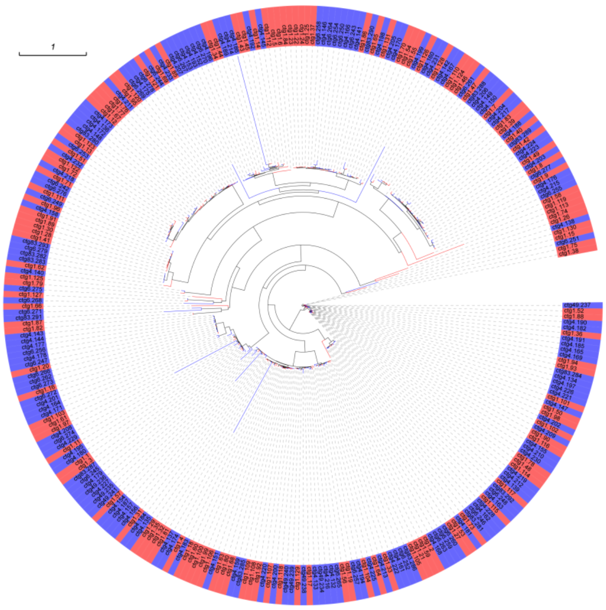

Figure S18. The phylogeny of 292 LINE/Rex-Babar of ctg1 and reciprocal sequence in the *Schizothorax o'connori* genome. The red color represented the transposon from R1 and

blue for R2. TEs with length < 200 bp were removed and the topology were inferred with Raxml (f a -m GTRGAMMA -# 100). Related to Figure 2.

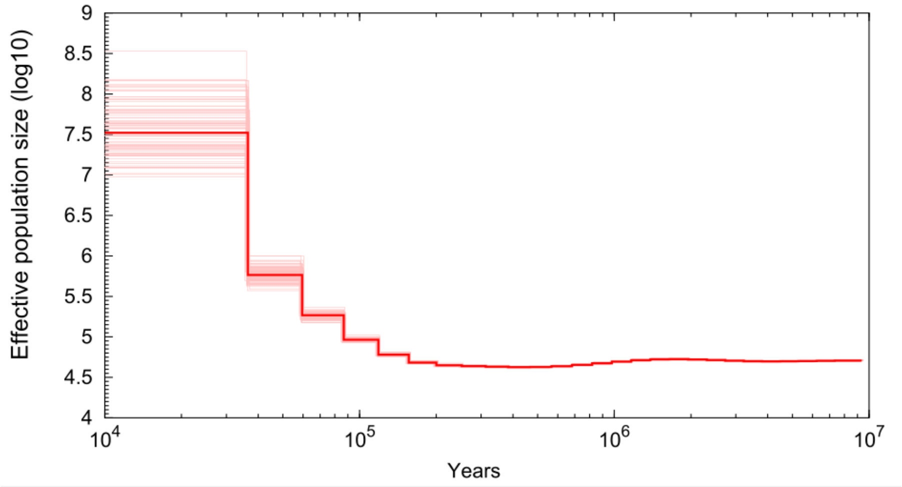

Figure S19 The deduced historical effective population for *Schizothorax o'connori*. Related to Figure 2.

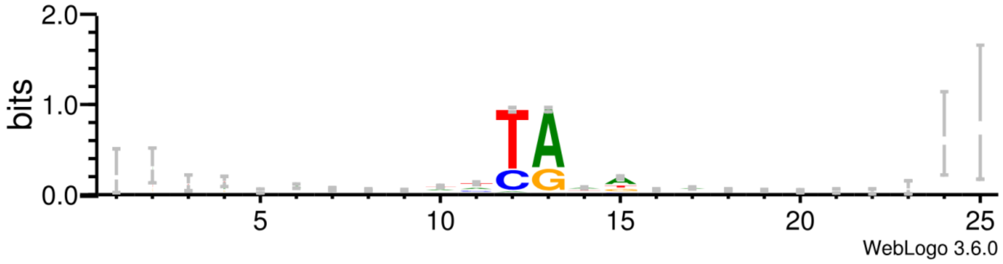

Figure S20 The webLogo show that "TA enrichment" around insertion sites between duplicated genomic regions. TA was the insert site of DNA transposons. Related to Figure 2.

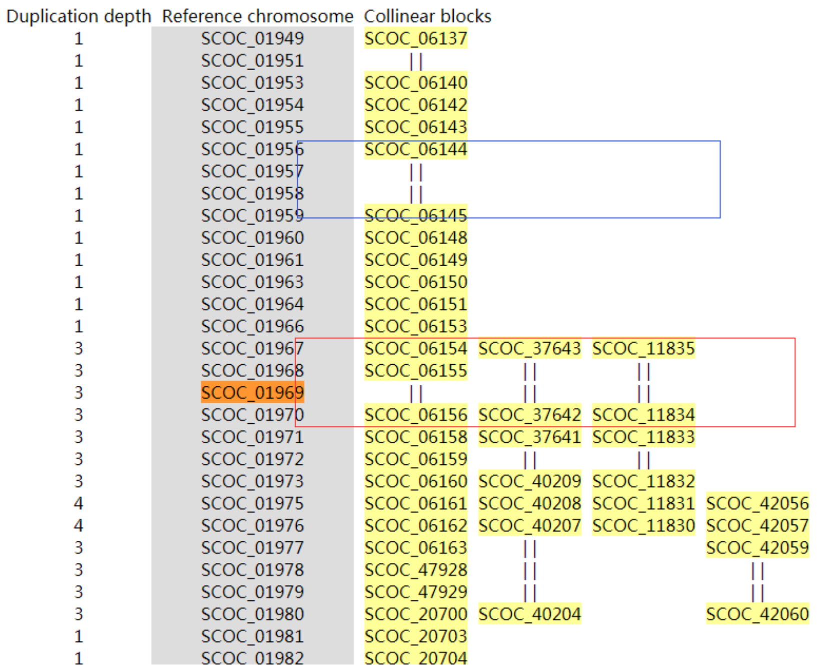

<table>
<tr><th>Duplication depth</th><th>Reference chromosome</th><th>Collinear blocks</th><th></th><th></th><th></th></tr>
<tr><td>1</td><td>SCOC_01949</td><td>SCOC_06137</td><td></td><td></td><td></td></tr>
<tr><td>1</td><td>SCOC_01951</td><td>||</td><td></td><td></td><td></td></tr>
<tr><td>1</td><td>SCOC_01953</td><td>SCOC_06140</td><td></td><td></td><td></td></tr>
<tr><td>1</td><td>SCOC_01954</td><td>SCOC_06142</td><td></td><td></td><td></td></tr>
<tr><td>1</td><td>SCOC_01955</td><td>SCOC_06143</td><td></td><td></td><td></td></tr>
<tr><td>1</td><td>SCOC_01956</td><td>SCOC_06144</td><td></td><td></td><td></td></tr>
<tr><td>1</td><td>SCOC_01957</td><td>||</td><td></td><td></td><td></td></tr>
<tr><td>1</td><td>SCOC_01958</td><td>||</td><td></td><td></td><td></td></tr>
<tr><td>1</td><td>SCOC_01959</td><td>SCOC_06145</td><td></td><td></td><td></td></tr>
<tr><td>1</td><td>SCOC_01960</td><td>SCOC_06148</td><td></td><td></td><td></td></tr>
<tr><td>1</td><td>SCOC_01961</td><td>SCOC_06149</td><td></td><td></td><td></td></tr>
<tr><td>1</td><td>SCOC_01963</td><td>SCOC_06150</td><td></td><td></td><td></td></tr>
<tr><td>1</td><td>SCOC_01964</td><td>SCOC_06151</td><td></td><td></td><td></td></tr>
<tr><td>1</td><td>SCOC_01966</td><td>SCOC_06153</td><td></td><td></td><td></td></tr>
<tr><td>3</td><td>SCOC_01967</td><td>SCOC_06154</td><td>SCOC_37643</td><td>SCOC_11835</td><td></td></tr>
<tr><td>3</td><td>SCOC_01968</td><td>SCOC_06155</td><td>||</td><td>||</td><td></td></tr>
<tr><td>3</td><td>SCOC_01969</td><td>||</td><td>||</td><td>||</td><td></td></tr>
<tr><td>3</td><td>SCOC_01970</td><td>SCOC_06156</td><td>SCOC_37642</td><td>SCOC_11834</td><td></td></tr>
<tr><td>3</td><td>SCOC_01971</td><td>SCOC_06158</td><td>SCOC_37641</td><td>SCOC_11833</td><td></td></tr>
<tr><td>3</td><td>SCOC_01972</td><td>SCOC_06159</td><td>||</td><td>||</td><td></td></tr>
<tr><td>3</td><td>SCOC_01973</td><td>SCOC_06160</td><td>SCOC_40209</td><td>SCOC_11832</td><td></td></tr>
<tr><td>4</td><td>SCOC_01975</td><td>SCOC_06161</td><td>SCOC_40208</td><td>SCOC_11831</td><td>SCOC_42056</td></tr>
<tr><td>4</td><td>SCOC_01976</td><td>SCOC_06162</td><td>SCOC_40207</td><td>SCOC_11830</td><td>SCOC_42057</td></tr>
<tr><td>3</td><td>SCOC_01977</td><td>SCOC_06163</td><td>||</td><td></td><td>SCOC_42059</td></tr>
<tr><td>3</td><td>SCOC_01978</td><td>SCOC_47928</td><td>||</td><td></td><td>||</td></tr>
<tr><td>3</td><td>SCOC_01979</td><td>SCOC_47929</td><td>||</td><td></td><td>||</td></tr>
<tr><td>3</td><td>SCOC_01980</td><td>SCOC_20700</td><td>SCOC_40204</td><td></td><td>SCOC_42060</td></tr>
<tr><td>1</td><td>SCOC_01981</td><td>SCOC_20703</td><td></td><td></td><td></td></tr>
<tr><td>1</td><td>SCOC_01982</td><td>SCOC_20704</td><td></td><td></td><td></td></tr>
</table>

Figure S21 Gene lost events displayed on synteny blocks. The blue rectangle means gene lost, for the red rectangle block, it may be caused by other whole genome duplication, so we don’t consider about it. Related to Figure 2.
